# Supplementary material for: Evaluation of oral health services and challenges faced by oral health practitioners working in Nyarugenge, Rwanda
Source: PLoS One. 2024 Aug 19;19(8):e0309127. doi: 10.1371/journal.pone.0309127 (PMC11332939; doi:10.1371/journal.pone.0309127)
Supplement: S1 Dataset — (ZIP) [file pone.0309127.s001.zip › dataset/Dataset qualitative interview transcript/PARTICIPANT (1).pdf]

INTERVIEW WITH PARTICIPANT 1

**Interviewer: Thank you for accepting that we have this interview. We shall try not to take a lot of time. As we told you, we are doing research related to the PhD of our colleague. He would like to know the challenges facing dental practitioners in rendering oral health services to the community. As you know, there is no wrong answer; all answers are correct and important. In addition, we assure you that the research is confidential; no one will be able to link the information you give us to you. Feel free and answer in any language you are comfortable with. We shall try not to keep you very long.**

**To avoid losing any information, we would like to record the voices. Do you give us that permission?**

*Interviewee: In which way will the record help you?*

**Interviewer: It will help us to capture every piece of information because it is impossible to write everything.**

*Interviewee: Ok. No problem*

**Interviewer: Thank you so much. Now, the first question goes like this. Can you tell us briefly the way you perceive your work? Are you happy with it? Is it really tiresome? Are there any challenges? Do you sometimes have to rash and work very quickly in order to clear the line? Feel free and tell us how it is.**

*Interviewee: Concerning my work, effectively, there are many patients, but we try to make plans to avoid being stressed. No one can be stressed because you receive the patients and examine them yourself, and plan how you will satisfy all of them in an equitable way. We have a system of giving appointments. We give rendezvous according to the number of patients we can treat. There are some patients who come and want that you treat them in a special way so that they receive their treatments the same day, while you already have those who came on appointment. So, the work cannot stress me because I am the one who gives appointments, but it depends on the period. There are some periods when patients are so many and other periods when children are at school and patients are few.*

**Interviewer: But apart from that, are you happy with your work?**

*Interviewee: Yes, I like my profession. When you like something, you are happy to do it well.*

**Interviewer: Were you expecting to receive such a great number of patients on a daily basis?**

*Interviewee: No, I was not expecting that because in Congo, people don't utilize dental services a lot. In fact, they don't have medical insurances like here. Here, everyone has the ability to come for dental care because of the community based health insurance (CBHI) and other insurance schemes.*

**Interviewer: And what effect did the fact of receiving many patients while you were not expecting it had on you?**

*Interviewee: I was not expecting that but when I came I was a fresh graduate, one year after completing dental studies. On our side we wished to receive many patients and when I came I was happy because I enjoy working.*

**Interviewer: Now, tell us about giving oral health education to all patients who come to you. Tell us, is it really possible? If it is not possible or if it is very challenging, can you explain to us why?**

*Interviewee: It is not possible to give oral health education to every patient. Most of the time we do it for patients with poor oral health status, or with halitosis; that is when we tell them that they have to brush their teeth, or again if the patient shows interest and asks you. Since we have many patients to examine and send them for the payment, especially that they spend a lot of time to the cashier, it is really challenging to find time to explain to the patient about routine oral healthcare, knowing that there are many patients waiting for you.*

**Interviewer: It means that you have the challenges linked to the number of patients and the processes of payment?**

*Interviewee: Yes, because on a single day you have patients for consultation, you send them for payments and wait for them. When they come back, you start treating. In between if there are other patients in the system you also see them. However, we try to work quickly in order to finish early. There is no time to start explaining about oral hygiene, we do it only when it is necessary for patients with periodontal problems like foul odor and a lot of calculus. We explain to them what to do in order to have a good oral hygiene.*

**Interviewer: When you manage to give oral health education to some patients, which are the main topics do you tell them about?**

*Interviewee: I explain simply the brushing technique for patients with gingival recession, I explain how to maintain a good oral hygiene.*

**Interviewer: When you give oral health education, do you have didactic materials?**

*Interviewee: No we don't have. We don't have typodonts and materials which would help us to explain the technique for dental brushing. When we do it only in theory, we are not sure of the outcome. Didactic materials should be available on our consultation tables but we don't have them.*

**Interviewer: Does it happen that you show the dental floss to the patient?**

*Interviewee: No, I don't have the dental floss, I explain only in theory. Once a medical delegate gave me one piece but I don't know where I threw it.*

**Interviewer: Meaning that the challenge you have in oral health education is lack of didactic materials?**

*Interviewee: Yes, we only give oral health education in theory but we cannot verify if they understood well or not.*

**Interviewer: You told me that you usually receive many patients with periodontal problems, do you think it is possible that you provide periodontal treatment to every patient who needs it the same day they came?**

Interviewee: No, it is not possible to do it the same day because every day there are patients on appointment and every day there are also patients for the consultation. We examine them and then fix a rendezvous for them for treatments.

**Interviewer: It means that every day you perform all types of treatments like tooth extraction, dental fillings …..**

*Interviewee: (interjecting). Yes, all are performed. For me I do all types of treatments in order to help the patients. When I don't have many patients, I do surgical extractions but normally in this wing I do endodontic treatments. Surgical extractions, and all types of fixations after accidents are dealt with in the maxillofacial wing. I do restorative treatments like endo and other fillings.*

**Interviewer: How many patients can you serve per day?**

*Interviewee: It depends on days. When patients are not so many, I do between eighteen and twenty-five consultations and when patients are many, I can do up to thirty consultations. For the treatments, I can do between ten or fifteen treatments per day.*

**Interviewer: It means that you alone can do consultations for twenty-five patients per day and fifteen treatments?**

*Interviewee: No, I don't work alone, I work with the others. After consultations I organize and we share the patients. Currently we have graduates in internship and I also work in team with one dental therapist. When interns are not there and that patients are many, one does the consultations while the other is treating.*

**Interviewer: Tell us about the sterilization of instruments, no problem on that side? Does it happen that you lack a sterile instrument for treating a patient?**

*Interviewee: It happens that we miss instruments for treatments because we don't have many instruments. On the side of the sterilization, we used to have an autoclave which functioned well but recently it got spoiled. We are obliged to send our instruments in the general sterilization department in the theater and we collect them every morning. We have a small autoclave in the dental service where we can put some instruments we need to be reused for other patients. They changed the cleaner recently and the new one tries to sterilize between treatments.*

**Interviewer: Meaning that currently the situation is somehow stable?**

*Interviewee: Yes, a little bit because we sterilize in a big container from the autoclave at the theater and in the morning, they bring them back and put them in small boxes.*

**Interviewer: Thank you so much. You told me that sometimes it is challenging to do oral health education before the treatment but, is it possible to give at least post-treatment instructions?**

*Interviewee: Yes, yes. Depending on the treatment I did either tooth extractions, root canal treatments, or crown reconstructions, I explain to the patient and provide appropriate advices on what they have to do. You cannot put an artificial thing in the mouth and fail to inform the patients, because some might ignore it and start to use the tooth as if it was natural.*

**Interviewer: Thank you so much. Tell us briefly about the quality of care that is provided here? How is it?**

*Interviewee: We try to do our best in order to give quality dental care but sometimes we lack a lot of materials.*

**Interviewer: Can you give us an example of what you wanted to do and which you were able to perform, but you failed to do it because of instruments or materials?**

*Interviewee: Sometimes we lack endodontic materials and we are obliged to call Muhima district hospital so that they don't send patients here but refer them to Rwanda Military Hospital. Sometimes there is shortage of materials even at the suppliers' side; we may even stop doing some treatments like root canal treatment and GIC filling due to stock out of materials. You cannot perform a treatment without materials even if you have the will to do it. However, currently those materials are available.*

**Interviewer: How long can it last without having necessary materials?**

*Interviewee: It is difficult to estimate but it can take a long time. In these instances, we inform people who use to refer patients here that we are not performing such or such treatment because we don't have materials. It can last many months without materials and when they bring, the stock is finished quickly because there were many patients on the waiting list.*

**Interviewer: And you stop again?**

*Interviewee: Yes*

**Interviewer: When one of the equipment like the dental chair, the compressor, or the sterilizer gets spoiled or is not functioning well, does the administration hurry up to repair it? How is it?**

*Interviewee: There is no problem about repairing. When there is something wrong on the dental chair, we call the technician who is appointed at our dental service and he/she comes immediately. When it happens that they are maybe on a night duty, they give you the telephone number of the other one who is available. There is no problem about repairing the dental chair, we have many technicians here.*

**Interviewer: Good. What about the sterilizer?**

*Interviewee: We had a big sterilizer which allowed us to sterilize everything from here. It got damaged and it is not yet repaired. Maybe technicians are not conversant with how to repair it. It is possible that only technicians who studied that domain can repair it.*

**Interviewer: Is it possible that they can do dental scaling and not polishing due to lack of polishing paste?**

*Interviewee: Yes, sometimes they only do scaling and not polishing because the polishing paste is not available. At times they try to use normal toothpaste for polishing.*

**Interviewer: Thank you (smiling). How secure do you feel when you are treating patients, especially on the side of the risk of contracting an infectious disease?**

*Interviewee: Yes, since I treat all kinds of patients even the ones suffering from chronic infectious diseases, I know that I must make sure to protect myself even when there is no apparent infectious disease and I take care not to injure myself. Elements for personal protection are available here unless the practitioner neglects to wear them. I use eye glasses, medical coat and gloves. They even brought face shields for use but I don't use them because they make me uncomfortable.*

**Interviewer: (both smiling). Do you sometimes use the cover rolls and other plastic covers for the light and the dental chair? Are they available here or not?**

*Interviewee: No, we don't have them.*

**Interviewer: Which advices can you give in order to make your job easier?**

*Interviewee: If I could get all the necessary equipment and materials for dental treatments, it could make my job easier.*

**Interviewer: What about the dental staff. Is the number enough? Would you wish that more staff are recruited?**

*Interviewee: Except maybe for the radiographer but otherwise I accomplish my duties and I don't have anything to say about recruitments. I know that the process of recruiting a radiographer is undergoing because if students were not there, no dental staff would be available for staying in the x-ray room, all of us are busy treating patients. Currently there is no problem about the number of clinicians, only for the x-rays room we are looking for one. The request has already been submitted and we wait for the decision from the administration of the hospital.*

**Interviewer: Coming back to the application we were talking about at the beginning, if there was an application which would be installed in patients' smartphones in order to**

**give oral health education in general, what importance that would have on your daily work?**

*Interviewee: If that application was installed for everyone, it can help us because this would reduce oral diseases. I think that this would help the population even those who don't want to attend dental services; they will have an idea on how to care for the teeth. The impact would be positive for dental professionals. However, I doubt because there are other applications on you tube where many dentists provide oral health education; but if one has not attended school, they cannot start searching you tube for oral health education sessions like on brushing techniques and others. If people search you tube, they can find what dentists are saying about the quality of a tooth, but if that application is installed everywhere, all the people will be informed about oral health and will know how to maintain a good oral hygiene. It will be very helpful and dental problems will be reduced especially for people living in remote villages. They have more dental problems due to lack of awareness. Since everybody can have access to the phone, all of them will have an idea on that.*

**Interviewer: Do you think that this application can reduce the time you used to spend with patients teaching them?**

*Interviewee: Yes, it will reduce that time because sometimes we don't even have time for oral health education. It will really help people. Someone may come for consultation and if he/she is smart, they can start asking how they can take care of their teeth or what is happening with their teeth. Only people who went to school ask such questions, looking for explanations about oral health. For the others, they come insisting that their teeth should be removed because they are painful; they don't know the value of the tooth.*

**Interviewer: How many instruments do you have for scaling? Do you use ultrasonic or manual scalers?**

*Interviewee: We use ultrasonic scalers. Manual scalers are also available but we use ultrasonic but there is a problem with scaler tips, they are few. We have requested them but we have not yet received. There are only two or three remaining.*

**Interviewer: Which advices can you give so that all the materials and equipment needed in teeth scaling and polishing are available and adequate?**

*Interviewee: Usually I don't know where they send the request either to KIPHARMA or other pharmacies. The dental service makes a request and send to the pharmacy of the hospital which*

*in turn sends it to the suppliers but sometimes you can miss instruments and materials for scaling even at the side of the suppliers themselves. We wonder if our pharmacists make a good follow up because we always make lists which are not honored. The problem is between our pharmacists and the suppliers.*

**Interviewer: Now the last question. I asked previously what you think might make your work easier. Let me now ask about the advices you can give in terms of equipment like the dental chair, the sterilizer or the x-ray machine, so that your work becomes much easier?**

*Interviewee: Concerning equipment, we only wait, there are staff responsible for doing requisitions. If the equipment is there, we also are there for treating the patient but if the equipment is not there we cannot treat by miracle. We are willing to treat patients like currently we still have few materials left. For the x-ray staff, we are waiting patiently but we know that the in charge of the dental department are already working on that. Otherwise, if interns were not there or other students in clinical placements, it can be challenging. We are normally two clinicians by wing, six dental staff in all, if the supporting staff is not there maybe every wing will start looking how to take x-rays for their own patients.*

**Interviewer: Let us hope that they will recruit that person and thank you for your collaboration. It was really very interesting and all the information is important for us. Thank you and nice work.**

*Interviewee: Ok. Thank you.*
