# Supplementary material for: Evaluation of oral health services and challenges faced by oral health practitioners working in Nyarugenge, Rwanda
Source: PLoS One. 2024 Aug 19;19(8):e0309127. doi: 10.1371/journal.pone.0309127 (PMC11332939; doi:10.1371/journal.pone.0309127)
Supplement: S1 Dataset — (ZIP) [file pone.0309127.s001.zip › dataset/Dataset qualitative interview transcript/PARTICIPANT (10).pdf]

INTERVIEW WITH PARTICIPANT 10

Interviewer: Thank you for accepting that we have this interview despite the fact that you are so tired, we shall try not to be long so that you might take some rest. As we told you, we are conducting a PhD research about the challenges dental staff are meeting in their daily work, but also the impact an application which would be put into the phone like they install WhatsApp and you tube, with the aim of educating people about oral health, would have on their work. What that application would be doing and what is its role in your work? As you know, research is confidential and every answer is important; there is no wrong answer. We would like that you answer freely and tell us everything. We are also requesting your permission to record your answers. Do you agree?

Interviewee: No problem

Interviewer: Thank you so much. Now, the first question goes like this. How do you perceive your work currently? Are you happy with it? Is it tiresome? Do you sometimes have to rash and work very quickly in order to clear the line? Are there any challenges? Feel free and tell us how it is.

Interviewee: I am happy with my job. The number of patients is higher compared to the number of dental practitioners. Sometimes you have to rush in order to clear the line of patients. Otherwise there is no problem except that sometimes materials are few and we are not able to provide a complete service. These are the only challenges I see here. The big number of patients compared to the number of clinicians and the lack of materials because sometimes we experience stock-outs for some of them.

Interviewer: Were you expecting to receive such a great number of patients on a daily basis?

Interviewee: Yes, I was expecting to receive many patients here.

Interviewer: Nothing surprised you?

Interviewee: What surprised me is irrelevant. I was surprised to see that there is no motivation. We need some kind of motivation in our work.

Interviewer: Now, tell us. Is it really possible to give oral health education to every patient who comes to you?

Interviewee: It may be possible when you have few patients or when you have started your work early. For others we give post-operative instructions related to the treatment we offered them. We also tell them how to brush their teeth. You cannot say that we have enough time to teach patient, there are still challenges.

Interviewer: It means that the challenge you have concerning oral health education is linked to the number of patients?

Interviewee: Yes

Interviewer: When you manage to give oral health education sometimes, which are the main topics do you tell them about? You told me that you tell them about tooth brushing, what else do you tell them about?

Interviewee: Focusing on the department in which I am appointed currently, we do surgical extractions and intermaxillary fixations (IMF). We tell the patient not to disturb the wound, and all necessary precautions to avoid post-extraction infection. No matter what, I must find time to give them these instructions.

Interviewer: But as you said that when patients are few or when you started earlier you can get some time for oral health education, which are the topics you tell them about?

Interviewee: No, I cannot get time to teach patients everything about oral hygiene; I cannot manage.

Interviewer: Now, when there are people who need scaling and polishing of teeth, what happens?

Interviewee: There is still a gap in the understanding of the necessity to look for dental cleaning. Many among patients we receive who had an accident or who need any other treatment have dirty teeth. It requires that we remind them and ask them to come for cleaning. The number of patients who come for that is still low.

Interviewer: Is it possible that you do scaling and polishing for every patient who needs it the same day they came?

Interviewee: No, there are many appointments and everything turns around what I already told you related to the number of patients, the insufficiency of materials and the shortage of clinicians.

Interviewer: Tell us now about the sterilization of instruments, how is it?

Interviewee: I see that they try. We have two small autoclaves. We use them and in cases instruments are many, they are placed in a drum and carried for sterilization in the central sterilization department of the hospital.

Interviewer: It means that you cannot fail to treat a patient due to lack of a sterile instrument?

Interviewee: We have few instruments; it requires that the sterilization personnel sterilize very often. When sterilizers are not available or when they are damaged, it becomes challenging for us.

Interviewer: Talking about scaling and polishing, how many patients can be treated per day, based on the instruments you have?

Interviewee: We currently have two or three scaler tips only, and it also depends on the chairs which has scaling apparatus. They are not enough compared to the number of patients.

Interviewer: Tell us briefly about the quality of care that is provided here? Are you happy with it? Is it really good? Are you satisfied?

Interviewee: No, the quality of care is still very low. I am not satisfied.

Interviewer: Why?

Interviewee: Seen the big number of patients coming here, there should be enough clinicians to receive them, so that they might get a comprehensive dental care, which would reduce clinicians overload. If there was a balance of dental materials, clinicians who receive those patients and patients themselves, I would be satisfied.

Interviewer: Apart from that, whatever you have materials for, do you provide it well and patients are happy?

Interviewee: Very well

Interviewer: When one of the equipment like the dental chair, the compressor, the sterilizer, or the x-ray machine gets spoiled or is not functioning well, does the administration hurry up to repair it? Do they neglect? How is it?

Interviewee: It depends on which one is spoiled. Here we have a good system; on every equipment there is a telephone number of the one we should call. There is a company here

called filos; when there is a machine which is spoiled or when the internet is not working, you call the number and inform them which room or which service needs their assistance. They immediately send someone to repair that. In addition, they also do maintenance; after a given period, they come and check all the equipment. When it is the compressor, we have technicians located here and who come to repair it. When it is beyond their abilities like for OPG, there is a man coming from KIPHARMA who comes and repair it; it cannot go beyond one day. They repair them quickly.

Interviewer: It means that they are valued?

Interviewee: Yes

Interviewer: Apart from the equipment, what about consumables?

Interviewee: The request of consumables is done on time but since we are in a public hospital, the process of delivery is very long so that at given times, we stop doing some procedures because dental materials are not there. That process causes us to experience stock-outs.

Interviewer: Is the polishing paste currently available?

Interviewee: Yes, we have like two full bottles. We have it in enough quantity.

Interviewer: And it is effective? Are stains removed?

Interviewee: Yes, it is. Stains are removed.

Interviewer: Concerning infection control, do you feel secure about the risk of contracting an infectious disease?

Interviewee: Yes. We have gowns and we make a requisition when they are about to finish. We also have face shields; we try.

Interviewer: What about the head?

Interviewee: For the head we don't have any protective equipment; we used to have them but currently there is a stock-out. All in all, infection control level is fair.

Interviewer: Now, what could be done in order to ease your work in general?

Interviewee: If they increase the number of chairs, we would share the patients and the work would be much easier.

Interviewer: Apart from chairs?

Interviewee: Consumables also. If they could be availed on time without stock-outs, it would be very helpful in our performance and patients would be satisfied.

Interviewer: I have seen someone assist you, do you always have assistants when you are treating patients?

Interviewee: No, even I was obliged to ask permission for that one to leave his wing and assist me. When students are not around, finding an assistant is very challenging.

Interviewer: Can you expand a little more on this?

Interviewee: This turns around what I told you above that here we need enough people to receive patients so that we might perform better.

Interviewer: I understand. Now, if there was an application which would be installed in patients' telephones in order to give oral health education in general, what impact that would have on your daily work?

Interviewee: This would help us a lot because patients would know how to perform oral hygiene; you can see some oral diseases and realize that they were caused by the lack of knowledge on the importance of oral health. That application is very important because it would reach where we couldn't.

Interviewer: Yes. Do you think that this application can reduce the time you used to spend with patients?

Interviewee: Yes. If we take the example of scaling, if patients were able to perform a good oral hygiene, with appropriate equipment, we wouldn't receive many patients with periodontitis or gingivitis. It would reduce the number of patients with problems caused by lack of knowledge.

Interviewer: You told me that there are only two ultrasonic scaler tips while patients in need of that treatment are many; which advices can you give so that all the materials and equipment needed in teeth scaling and polishing are available and adequate so that you might provide a better service?

Interviewee: The reason why we have two scaler tips is because there are two ultrasonic scalers. When you need to do scaling, you ask your colleague to switch so that the patient who needs scaling would be installed on the chair with ultrasonic scaler. If all the chairs had

ultrasonic scalers and enough scaler tips, this would ease our work and it would be helpful for patients.

Interviewer: Is the ultrasonic scaler coming without scaler tips?

Interviewee: It has but as you know, this is a teaching hospital where students may come and while using it, they close incorrectly. That would spoil the tip and the process of getting new ones is very long as I told you before; by the time of the next delivery, what we had would already be finished or no longer functioning. That hinder us in our desire to help patients.

Interviewer: It is understandable. What would ease your work in general? Which advices could you give on that?

Interviewee: To be at ease, I would need dental materials and an assistant.

Interviewer: Assistants and materials?

Interviewee: Yes

Interviewer: Thank you so much, kindly forgive us because we made you hungry.

Interviewee: There is no problem.

Interviewer: And we thank you for the information you gave us, it is very important.

Interviewee: Thank you too.
