# Supplementary material for: Evaluation of oral health services and challenges faced by oral health practitioners working in Nyarugenge, Rwanda
Source: PLoS One. 2024 Aug 19;19(8):e0309127. doi: 10.1371/journal.pone.0309127 (PMC11332939; doi:10.1371/journal.pone.0309127)
Supplement: S1 Dataset — (ZIP) [file pone.0309127.s001.zip › dataset/Dataset qualitative interview transcript/PARTICIPANT (11).pdf]

INTERVIEW WITH PARTICIPANT 11

Interviewer: Thank you for accepting that we have this interview. We are conducting a PhD research about the challenges dental staff are meeting while treating Nyarugenge population but also the impact an application which would be put into the phone in educating patients about oral health would have on their work. In research there is no wrong answer, every answer is good, correct and important. You also know that every information is kept with confidentiality, no one will know who said this or that. We would like that you answer freely and we are requesting your permission to record your answers so that we don't lose any information. Do you agree?

Interviewee: I agree

Interviewer: Thank you. The first question is this one: H**ow do you feel about your work currently? Are you pleased to do that job? Is your job tiresome? Do you sometimes have to rash and work very quickly in order to clear the line? Are there some challenges? Tell us about how it is.**

Interviewee: I am happy with my job currently because this is a profession that I joined willingly. In addition, I have exercised it for quite long so that I feel like immersed in it and I plan to keep exercising it and receive well my clients.

Interviewer: What about that aspect of being tiresome?

Interviewee: Telling the truth, the work is not tiresome when you have adequate materials and equipment like the dental chair from which you can change the position of the patient as you wish.

Interviewer: It means that when treating patients, you feel relaxed and calm, without rushing in order to clear the line? How is it?

Interviewee: Currently I am not rushing to clear the line because the number of clinicians is enough when compared to the number of patients we receive. We are not so overloaded.

Interviewer: Yes. Now, when patients come to you, are you able to give them oral health education individually?

Interviewee: We teach them but it is not enough. Educating them on the chairside is not enough because there is a lot of information they need to know which cannot be entirely delivered here, so that they might be able to take care of their teeth and to know that they should come on time

for treatment. Another challenge is linked to materials we need for treatments. Apart from the equipment which itself is not enough nor fully functioning, dental materials like the ones we use for dental fillings are very rare on the market. In public hospitals it is even worse because the process for getting them is not easy. That is the biggest challenge we meet. If for example you have only one dental chair from which you can do dental fillings, even when it is five patients waiting for it, their waiting time will be long which would not be the case if they could be treated from two or three working stations. Unfortunately, equipment and materials are still a challenge as I told you.

Interviewer: You told me that you try to do oral health education to patients even if it is not comprehensive; what are the main topics that you tell them about?

Interviewee: The first topic we talk about is oral hygiene; how to brush teeth and how to do regular check-ups for advice on oral health, that is it briefly. After treatment, we give them post-operative instructions related to what we did for them. When you did an extraction, you tell them how to avoid complications; when you did a filling, you give instructions on how to take care of the filled tooth so that the filling doesn't break and that it is not dislodged.

Interviewer: When you are giving that oral health education, do you have didactic materials to use in order to show the best way of brushing teeth or you tell them only in theory?

Interviewee: Most of the time, we tell them in theory

Interviewer: Due to lack of didactic materials or why?

Interviewee: Didactic materials are few. We have only one dental model and one tooth brush, you cannot use it every year and even those we have belong to the students. For us, there is no way you can make a request for didactic materials.

Interviewer: Now, tell us about scaling of teeth. Is it possible that you provide that treatment to every patient who need it? Are there many patients who need it? How is it? Tell us about it.

Interviewee: Many are in need of it but most of the time they are not aware of it. Sometimes we clean them while they came looking for a tooth extraction. They often come complaining of tooth pain but they don't know the cause of that pain. When you check, you can find that what is needed is scaling. Sometimes you find that they have a carious tooth which needs to be filled

but when you check the oral cavity you find that at the same time they have calculus. You can do the filling and fix a rendezvous for scaling or you can even do both on the same day.

Interviewer: Do you also do polishing after scaling?

Interviewee: Yes, we do it

Interviewer: How many patients can be treated by scaling and polishing per day, based on the number of instruments you have?

Interviewee: We can do scaling and polishing for at least five patients per day, changing scaler tips.

Interviewer: Do you have five scaler tips?

Interviewee: Yes, we have them

Interviewer: Tell us now about the sterilization of the scaling instruments. How is it?

Interviewee: We sterilize instruments according to current standards. Those standards are set in hospital's policies. There is a policy related to sterilization of instruments. We sterilize from the main sterilization unit of the hospital.

Interviewer: You don't have a sterilizer here in the dental service?

Interviewee: No, it is not allowed.

Interviewer: Really? It means that the one you used to have here in the service has been moved to that common sterilization unit?

Interviewee: Yes of course, especially that what we had was a dry heat sterilizer and currently they are using autoclave.

Interviewer: And it doesn't disturb you in any way about getting sterilized instruments?

Interviewee: The way it disturbs us is that in the main sterilization unit, instruments coming from all hospital services are collected there; sometimes we can miss sterilized instruments because they bring them late, thus increasing the patients waiting time. When those machines get damaged or when the water used in the sterilization process is not there, it is a big challenge. Fortunately, it doesn't happen very often but it delays our work.

Interviewer: What machines were you talking about?

Interviewee: The sterilization machines

Interviewer: You told me that after treatment you give instructions, that is good, but when you think about the quality of care that you provide in your dental service how do you feel about it? Are you happy with it? Should it be improved? How is it?

Interviewee: We are happy with it but not fully. If you remember what I told you before, the quality of care has a component of customer care. If a patient comes here and you lack materials for treating him/her, you refer them to hospital x and arrived there they may also find that the materials are not available. They tell the patient to come back here so that they refer them hospital y because hospital x cannot refer to hospital y, such cases are so many. Our quality of care is handicapped by the fact that we cannot get dental materials in an easy way. If dental materials were available, this would be alright. I don't know exactly what you meant about quality of care but for me I understand it in view of customer care and skills of clinicians.

Interviewer: That is what I was about to ask

Interviewee: On the side of clinicians, considering our scope of practice, we are satisfied of the service we offer.

Interviewer: Are the patients also satisfied with what you do for them?

Interviewee: Yes, patients are very happy especially that we display a good customer care. The patients even talk about it and write about it in the suggestion box, telling other clinicians to learn customer care from the dental service.

Interviewer: When one of the equipment gets damaged like the dental chair, the sterilizer, and the compressor, do you have an x-ray machine?

Interviewee: The x-ray machine is there but the developed is not functioning. It was spoiled for quite a long time until our dental films got expired. We have requested self-developing dental films in order to come out from that routine but the process of delivery is also not easy. For the dental chairs, only one is fully functioning. It has an ultrasonic scaler as well as high and slow speed handpieces.

Interviewer: When one those important machines gets spoiled, does the administration hurry up to repair it? Do they give value to them?

Interviewee: That is where I was coming. I want to express it from the central level, meaning from the Ministry of Health. The way dental service is given priority is not good. That appears clearly when you do a requisition of materials especially at the hospital level. They cannot understand that the dental service is also generating money or that it is important because it serves the population. The priority is given to the maternity, pediatric and neonatology services. The dental service lags behind. You cannot convince someone to buy an equipment for dental service when there is one needed for the maternity service. We always wonder if the dental service is futile or is not generating money so that its revenues cannot help to buy a scaler? If a scaler has been functioning for like two years, it is not understandable that the money generated during that period cannot buy another one in order to replace the damaged one. That is always a challenge. The administration doesn't value the service of dentistry. On our side we know that it is very important but when they think from the hospital box, it is not valued.

Interviewer: Is that why the other dental chair got spoiled and didn't attract their attention?

Interviewee: They don't care; it is not urgent or a priority for them.

Interviewer: Apart from the equipment, are consumables given priority?

Interviewee: That is also a problem. That is what I was saying. Maybe here at the hospital level, they can make the request of composite or amalgam because we put these items on our list of needs but when they send it to the pharmacy of the district, there they can find that the only consumables which are available in their store are dental needles and dental cartridges. The store manager of the hospital once told me that the only dental items she knew were dental needles and dental cartridges. At the district level it is even worse. Currently they have initiated a company charged to provide medicines and materials in all hospital services but you find that dental has been forgotten. Another thing, even when they provide, usually they bring different item from the one you requested. As a recent example, we requested for drilling burs and they brought polishing burs while we needed inverted cone bars.

Interviewer: Because they don't know them. They cannot even ask you to go with them so that you verify them before buying?

Interviewee: No, they don't involve us. They cling to their position of managers but they never request our expertise. Most of the time when we ask for an item, they bring a totally different one, from what they searched on google.

Interviewer: (both smiling). Do you have the polishing paste?

Interviewee: Yes

Interviewer: Is it effective?

Interviewee: Yes, it is effective.

Interviewer: Does it remove stains properly?

Interviewee: Very well. After finishing to clean patients' teeth they often don't recognize themselves.

Interviewer: Wonderful! How is the infection control when you are treating patients? Aren't you fearing that you might contract a disease while treating? How secure are you on that side?

Interviewee: Currently we have personal protective equipment like face shields, face masks, head caps, shoes, and disposable gowns so that when you are going to do a polishing you are really covered; nothing can harm you from the patient.

Interviewer: You are covered in that area

Interviewee: Yes, they bought these shoes for us when we requested them.

Interviewer: Now, what would ease your work in general?

Interviewee: The thing that would ease my work is the timely availability of dental materials. As an example, it has been more than a year without composite. We were using the one brought by students.

Interviewer: The one I once brought you? You managed it really well if you still had it.

Interviewee: Talk about zinc oxide eugenol; it is really hard to get it. This is common for all dental materials, getting them is really a big challenge. If they would allow us to get them in an easy way. We have made more than twenty requisitions but in vain. That affects the patients flow, from here to Hospital x, from hospital x back here, from here again to Hospital y; this is really sad for the patients and it is really stressful for us.

Interviewer: I understand. If there was an application which would be installed in patients' smartphones in order to give oral health education in general, what importance that would have for you?

Interviewee: That application would be very useful. That would be an innovative way of caring for oral health. I don't know the person who would install it in these smartphones but this would be a big contribution of the application owner in oral health promotion.

Interviewer: Which impact that application would have on your daily work?

Interviewee: If a person is informed about his/her teeth and how to care for them in case they get diseased, treating them would be easy because some patients might stay home, unaware that they need dental care. Even our colleagues' health professionals often don't care about their oral health. You can find someone with a dental disease but despite that we are working in the same hospital and we are together on a daily basis, they don't take the initiative to come to us. Most of the time they are not aware about the problem. That application would raise the awareness on different aspects of oral health. People to know that they are diseased and that they should go for treatment. For me I think that application would be very good.

Interviewer: How would that application help in your daily activities?

Interviewee: People would get a place where they would receive a comprehensive oral health education because what we offer is really summarized.

Interviewer: Do you think that this application can reduce the time you used to spend with patients teaching them? What can you tell us about that?

Interviewee: Yes, that time would be reduced. It is true that we are also required to educate patients while treating them, to give them post-treatment instructions and even to tell them about safe use of medications, like when you prescribe a mouthwash you should tell them how to use it. That application would do some of our work; you would complement it only by telling patients about what is not covered in the application, because you know that the oral health education they will get from the application is of good quality. Our time spent educating patients would be reduced, gaining more time for the treatment.

Interviewer: Yes. Now, which advices can you give so that all the materials and equipment needed in teeth scaling and polishing are useful for you?

Interviewee: Advices to the manufacturers or to who?

Interviewer: No

Interviewee: To the suppliers and our managers?

Interviewer: Yes.

Interviewee: The advice that I would give to our managers is that they would have a dental practitioner in their team. When you request for a scaler tip, whoever is not knowledgeable would go and buy only very thin ones and you would miss what to use for some areas; it needs someone who knows the different types of scaler tips. If they buy scalers of good quality, the clinician will be comfortable when using them.

Interviewer: Now, thinking about your job in general, which advices can you give in order to make it easier?

Interviewee: What I can say is that if patients who come to us knew about oral health, if you tell them that their teeth need dental filling or that their overall oral hygiene requires professional dental cleaning, they wouldn't be arguing and insisting that their teeth should be removed. That is where that application would play a role, in raising the awareness of our clients about oral health. The communication between dental patients and dental practitioners would be smoother. That wouldn't take us a lot of time trying to convince them about the best treatment.

Interviewer: Thank you. I think we talked about everything. You told me that you have enough staff and your need of dental materials. That is what we wanted to ask you. The information you gave us is very important and will be useful in our research. Thank you so much.

Interviewee: Thank you too.
