# Supplementary material for: Evaluation of oral health services and challenges faced by oral health practitioners working in Nyarugenge, Rwanda
Source: PLoS One. 2024 Aug 19;19(8):e0309127. doi: 10.1371/journal.pone.0309127 (PMC11332939; doi:10.1371/journal.pone.0309127)
Supplement: S1 Dataset — (ZIP) [file pone.0309127.s001.zip › dataset/Dataset qualitative interview transcript/PARTICIPANT (12).pdf]

INTERVIEW WITH PARTICIPANT 12

Interviewer: Thank you for accepting that we have this interview. We are conducting a PhD research about the challenges dental staff are meeting while treating patients but also the impact an application which would be put into the phone for educating patients about oral health would have on their daily work. We shall try not to take a lot of time. In research there is no wrong answer, every answer is important. We would like that you answer freely and we are requesting your permission to record your answers so that we don't lose any information. Every information is kept with confidentiality and will be used only for research purpose. Do you agree for recording?

Interviewee: Yes

Interviewer: Thank you. The first question is this one: How **do you feel about your work currently? Are you pleased to do that job? Is your job tiresome? Do you sometimes have to rash and work very quickly in order to clear the line? Are there some challenges? Tell us about how it is.**

Interviewee: Until now my work is good, it is not stressing me.

Interviewer: It is not even tiresome?

Interviewee: It depends on the number of patients you received. When they are many you have to work hard in order to receive them all and offer them a good service. In such occasions you can be tired but when the number of patients is fair, there is no problem.

Interviewer: Does it happen very often that you receive a big number of patients?

Interviewee: Nowadays it is not very often because even health centres have their own dental therapists. They refer few patients to us especially for dental fillings or scaling. Briefly, the work is no longer tiresome.

Interviewer: It means that since you don't have a lot of patients, you can provide oral health education to every patient who comes to you?

Interviewee: Yes. Since the number is not so big, whatever is needed we do it for them, when it fits into our scope of practice.

Interviewer: It means that for every patient you receive, you start by giving them oral health education?

Interviewee: Yes

Interviewer: Which are the main topics that you tell them about?

Interviewee: You mostly insist on their chief complaints. If they came for dental scaling, you would tell them about the importance of dental cleaning, you explain to them how they should perform oral hygiene, reminding them that it is very important because it allows to protect other teeth from diseases. If they came for dental extraction, you tell them the level of dental disease which necessitates to be treated by extraction and when it didn't reach that level, you advise them to opt for dental filling. You start by the chief complaint and add other things.

Interviewer: Yes. When you are giving that oral health education like when you are showing them the best way of brushing in order to protect teeth, do you have didactic materials to use or you tell them only in theory?

Interviewee: We tell them only in theory

Interviewer: Which challenges do you meet in that area of oral health education?

Interviewee: The first challenge is that absence of didactic materials, hindering you from doing some demonstrations on how to brush. When it is someone who is curious and wants to learn more, they ask you questions and you try to show them through gestures. There are other patients who seem not to understand what you are telling them; they are only listening.

Interviewer: Now, tell us about scaling and polishing of teeth. Is it possible that you provide that treatment to every patient who need it? Are there many patients who need it? How is it? Tell us about it.

Interviewee: Usually whoever comes requesting scaling, we do it for him/her. There are other patients who come for another treatment for whom we see that they also need scaling and polishing. In this case, we explain to them, tell them the advantages of it and fix a rendezvous for them. When they come back, you realize that they learnt from what you told them. They tell you that they used to have dirty teeth which made them uncomfortable but they didn't know the reason and what to do about them. When they come back, it shows that they are willing to change, because some of them neglect what we told them and they never come back.

Interviewer: Does it mean that you do it for all the patients who need that treatment on the day they come?

Interviewee: Depending on the instruments, we can't but we treat the majority of them.

Interviewer: You can treat like how many per day by doing scaling and polishing?

Interviewee: We can treat like five patients per day.

Interviewer: It means that you have instruments for only five patients?

Interviewee: No, it is not because we have instruments for only five patients but as you know, this is a long procedure and there are other treatments. If the five patients for scaling are added to five more for dental filling, and others for extractions, it takes a long time; that is why we have to limit.

Interviewer: How many patients can you receive per day, in general?

Interviewee: Dental patients?

Interviewer: Yes

Interviewee: When they are many, you can even go up to thirty patients per day.

Interviewer: Tell us now about the sterilization of the scaling instruments. How is it?

Interviewee: After using instruments on a patient, we soak them in a bucket with water and after finalising all the treatments, we put them into the disinfectant which is chlorine; afterwards we clean and dry them and then we carry them to the central sterilization unit.

Interviewer: You don't have your own sterilizer here in the dental service?

Interviewee: No

Interviewer: You can never fail to treat a patient due to lack of a sterilized instrument?

Interviewee: If we miss an instrument, we go back and sterilize it again but it rarely happens.

Interviewer: When you finish to treat a patient what happens?

Interviewee: When we finish the treatment we give post-treatment instructions based on the treatment we performed then we discharge them, telling them how to collect back their insurance and identity cards.

Interviewer: It means that you never lack time for post-treatment instructions?

Interviewee: No, we don't lack it

Interviewer: When you think about the quality of care that you provide in the dental service in general, how do you feel about it? Are you satisfied with it? Could it be improved?

Interviewee: If possible it should be improved. You cannot say that you are satisfied with the quality of care because there are periods when we experience almost a total shortage of dental materials. In addition, we would be happy if more dental staff were added especially dental surgeons in order to reduce references. If doctors were there and materials were available, cases we used to refer would be managed here instead of always telling the patients that there is no material or that there is no specialist to deal with their cases.

Interviewer: When one of the equipment gets damaged like the dental chair, the sterilizer, and the compressor, those important machines like x-ray's; how is it? Does the administration hurry up to repair it? What value do they give it when you inform them about that?

Interviewee: Almost none

Interviewer: Sure? Why?

Interviewee: Because managers often say that there are no emergencies in dentistry, they cannot buy an expensive equipment while maternity and paediatric also need some. Dental service always lags behind in the process of supplying. They don't consider it as a priority. That is also the case for consumables. We can make a request and wait for a whole year before receiving them. We always make a follow-up in writing, they tell us to wait. Sometimes they tell us that dental filling materials are nowhere to be seen in the district pharmacies and they are not allowed to buy outside these pharmacies. In such cases, we have to wait until the district purchase them.

Interviewer: The process is long indeed

Interviewee: It is long

Interviewer: If the only chair which is fully functioning came to be damaged, they would also delay the repair?

Interviewee: One chair has been damaged and they neglected but the one functioning has been bought by Rwanda Biomedical Centre and when there is a problem on it, they send a technician to check.

Interviewer: They don't delay?

Interviewee: No, since it is still in the guaranty period. We don't know what will happen when that period will be expired. Maybe we will have to wait again a long time.

Interviewer: I understand that equipment and consumables are still a challenge. Do you have the polishing paste?

Interviewee: We still have it for now but we don't know what will happen when it gets finished. It is not easy to find it.

Interviewer: Tell us about infection prevention when you are doing treatments. How is it?

Interviewee: Nowadays we have personal protective equipment. We have face shields, gowns, shoes, face masks, and gloves.

Interviewer: It means that when you are doing treatments, you feel secure?

Interviewee: Yes

Interviewer: You even have head caps?

Interviewee: Yes

Interviewer: Now, what could be done in order to ease your work in general?

Interviewee: The first thing which should be done is to avail dental materials on time, as soon as we make a request, because when they finish and that you have to wait for months or for a whole year, we get discouraged. We wonder if our administration really knows the importance of this service. Do they value it or do they at least value people who come for it?

Interviewer: It means that you have only what you are using here in the service, with no reserve in the hospital pharmacy?

Interviewee: Sometimes they buy like five small bottles of glass ionomer and one lasts two weeks' maximum. You understand that the five bottles will last two to three months. When they get finished we would have to wait like a whole year before getting that material again. That is so discouraging.

Interviewer: Yes. Any other thing that would make your job easier?

Interviewee: When materials are available, it is fine

Interviewer: If there was an application which would be installed in peoples' telephones in order to give oral health education in general, how to take care of oral health, what impact that would have on your daily work?

Interviewee: The application would have a big impact because people suffer from oral and dental diseases because they lack knowledge about that. Sometimes they suffer from dental pain but they stay at home by fear of tooth extraction which would cause them to be edentulous. If they were aware that a tooth can be filled, the would come early and get the filling which would allow them to keep their teeth. Due to that lack of knowledge, they stay at home and come at a late stage when nothing else can be done to save the tooth. If they are suffering from periodontal diseases, they keep wondering what these deposits are and what will happen. They don't know that they are harmful for the gum, that they can be removed, or how and when to perform oral hygiene; but if they have that application in their telephones, they would be aware of any problem on their teeth and hurry up to the dental practitioner.

Interviewer: Do you think that this application can reduce the time you used to spend with patients?

Interviewee: It can help us because when you are explaining something to the patient for the first time, it requires a lot of strength but in case they would have some information about that, it would be easier for us. They would understand quickly what you tell them, they would know the advantages of following your advices and the disadvantages of not following them. But when they hear that for the first time, it is very hard for them to catch anything. Sometimes when patients come back for another visit and that we ask them if they put into practice what we told them, they reply that that day they were not hearing anything, meaning that they didn't understand the first time. Only people with multiple dental visits understand quickly what you tell them, more than the one who comes for the first time.

Interviewer: Now, which advices can you give so that all the materials and equipment needed in teeth scaling and polishing are useful for you?

Interviewee: The first thing I can say is to avail dental materials and equipment; to replace them in case they get spoiled or to purchase what got finished. Secondly, if it was possible to provide mass oral health education, people would be aware about oral health, they would seek dental treatment very early before the teeth become hopeless.

Interviewer: Are the instruments and materials you use in scaling and polishing performing well. Don't you have some complaint about them?

Interviewee: It is only nowadays. The problem we have, they can be available this month and absent in the following month. Nowadays we have them and there is no complaint about them.

Interviewer: It means that for you, if materials were available, your work would be enjoyable?

Interviewee: Availability of materials and mass oral health education and promotion in the community. People would be aware of when and how to perform oral hygiene and consequences of poor oral hygiene. If there were sessions of oral health education within the community, people would come for treatment on time and they would come asking for professional dental cleaning.

Interviewer: Thank you. That is what we wanted to ask you. The information you gave us is very important and will be useful in our research.

Interviewee: Yes

Interviewer: Thank you so much.

Interviewee: Thank you too.
