# Supplementary material for: Evaluation of oral health services and challenges faced by oral health practitioners working in Nyarugenge, Rwanda
Source: PLoS One. 2024 Aug 19;19(8):e0309127. doi: 10.1371/journal.pone.0309127 (PMC11332939; doi:10.1371/journal.pone.0309127)
Supplement: S1 Dataset — (ZIP) [file pone.0309127.s001.zip › dataset/Dataset qualitative interview transcript/PARTICIPANT (13).pdf]

INTERVIEW WITH PARTICIPANT 13

Interviewer: Thank you for accepting that we have this interview. We are conducting a PhD research about the challenges dental staff are meeting while treating Nyarugenge population but also the impact an application which would be put into the phone for educating patients about oral health would have on their work and on oral health in general. In research there is no wrong answer, every answer is important. We would like that you answer freely because research is confidential, no name will be recorded. We are requesting your permission to record your answers so that we don't lose any information.

Interviewee: Yes

Interviewer: Thank you so much. The first question is this one: How **do you feel about your work currently? Are you pleased to do that job? Is your job tiresome? Do you sometimes have to rash and work very quickly in order to clear the line? Are there some challenges? Tell us about how it is.**

Interviewee: Thank you. Concerning how it is with my job currently, I am happy with it because I joined this profession because I liked treating oral and dental diseases. Until now I enjoy my work. The challenges we meet in receiving patients of Nyarugenge district are related to the role of the hospital management in availing dental materials. They don't supply us with dental materials on time so that we might serve our patients in a good and satisfying way. On the other side, there are many aspects of oral diseases that patients are not aware of. Many don't know about that; it requires us to keep teaching them.

Interviewer: But is it not very tiresome?

Interviewee: Currently I cannot say that it is tiresome. It used to be tiresome when there were still few dental practitioners. At that time our work was really tiresome but now it is no longer wearisome.

Interviewer: Now, are you able to give oral health education individually to every patient who comes to you?

Interviewee: Yes, whoever comes here, either during consultation or during treatment, we give them oral health education especially on oral hygiene. You cannot say that you lack time to do it. It can be challenging to have spare time exclusively for oral health education but since we

keep educating them from the consultation throughout the treatment, at the end you have done a deep oral health education.

Interviewer: What are the main topics do you tell them about during oral health education?

Interviewee: We focus mainly on oral hygiene. Many people don't know how to perform oral hygiene. I also tell them about oral and dental diseases. I cannot forget to tell them about harmful effects of canines' mutilation because many believe in these myths but you try to change their mind.

Interviewer: Don't you face challenges when you are doing oral health education?

Interviewee: Challenges are there especially because we don't have didactic materials. There are also some people who don't want to hear what you are telling them, thinking that you are wasting their time while you wanted them to acquire some awareness.

Interviewer: Now, tell us about scaling and polishing of teeth. Is it possible that you provide that treatment to every patient who need it? Are there many patients who need it? How is it? Tell us about it.

Interviewee: Yes, patients for scaling and polishing are many but when they come, we try and do it for them except when we are obliged to fix a rendezvous for another day; but most of the time we do scaling and polishing as patients come.

Interviewer: It means that there are no challenges in that area, whoever needs scaling and polishing you do it for them?

Interviewee: Yes, we do that because we have what is needed to do it.

Interviewer: How many patients can be treated by scaling and polishing per day, based on the number of instruments you have?

Interviewee: We have like five scaler tips.

Interviewer: And you don't sterilize them again during the day? You sterilize only once, at the end of the day?

Interviewee: Yes, we sterilize only once at the evening because the sterilization has been centralized for all the hospital departments.

Interviewer: It cannot happen that you send a patient back home because there are no sterilized instruments?

Interviewee: Nowadays, since patients are no longer so many, we sterilize only once and we don't lack sterile instruments to use for treatment. Before, we used to sterilize in between, when dental therapists were not yet appointed at health centers. Unless it is an instrument that we don't have, for which we have made a request but which is not yet supplied; but for those we have, no problem.

Interviewer: Those the ones you don't have, it is clear; but on the side of the sterilization, is everything ok?

Interviewee: The only problem we have in the sterilization area is that sometimes we get sterile instruments a little bit late.

Interviewer: It means that they don't sterilize instruments in the evening or at night and wait to do it the next morning?

Interviewee: They sterilize them in the morning but on our side we have some reserve from the previous day which we use before they bring the other ones.

Interviewer: Let us now consider that you have just finished to treat a patient, do you have time to give instructions related to the treatment offered to them?

Interviewee: Yes, we do it. For example, when you did a dental filling or a tooth extraction, you give post-operative instructions. If you fail to give those instructions, whatever you did for them would be useless.

Interviewer: It means that you find that time no matter what?

Interviewee: Yes, we find the time no matter what. A patient cannot go home without post-treatment instructions.

Interviewer: When you think about the quality of care that you provide in your dental service how do you feel about it? Are you happy with it? Should it be improved? How is it?

Interviewee: I wish it should be improved. If they could give us enough materials, and bring also dental surgeons so that more services would be offered from here. Patients would receive a bigger package of dental care form here; references would be reduced.

Interviewer: But considering what you are allowed to do based on your scope of activities, are you happy on how you deliver them?

Interviewee: On my side, I am happy of the way I deliver them.

Interviewer: When one of the equipment gets damaged like the dental chair, the sterilizer, and the compressor, does the administration hurry up to repair it? Do they give value to them?

Interviewee: Sometimes they delay to repair like the dental chair but nowadays they have somehow improved in collaboration with Rwanda Biomedical Center. They signed an MOU with the hospital and when we call them, they send a technician to repair it.

Interviewer: Apart from the equipment, are consumables like the polishing paste given priority?

Interviewee: For consumables it is even worse; they delay due to how the hospital request for materials and the company which replaced CAMERWA. It is still challenging.

Interviewer: When you are doing treatments, do you feel secure especially about the risk of contracting an infectious disease?

Interviewee: People have been aware about personal protective equipment (PPE) and we try to protect ourselves and to protect the patient we are treating.

Interviewer: Do you have enough PPE?

Interviewee: Yes, we have enough PPE especially because of COVID outbreak.

Interviewer: You cannot lack any given PPE item?

Interviewee: We have the full PPE.

Interviewer: Now, what would ease your work in general?

Interviewee: Better than we used to perform?

Interviewer: Yes. You told us that currently you are no longer overloaded because the number of patients reduced; however, you may wish for something else in order to work more comfortably.

Interviewee: What would ease my work is the timely availability of dental materials. A patient may come having a tooth which needs a dental filling; unfortunately, there is no appropriate

dental material to do it and you refer him/her to Hospital x. If that situation was corrected so that we would get what we need to use on time i.e. dental chairs, instruments and dental materials, we would be very happy. Delivering materials in public hospitals is a big challenge.

Interviewer: If there was an application which would be installed in patients' telephones in order to give oral health education in general, what impact that would have on your daily work?

Interviewee: The impact of that application would be very positive because the patient would be informed about oral health. Most of the time when you tell something to the patients, it is new for them. But if they had seen it somewhere else like on the telephones, they would be aware of some topics and it would be easy to explain to them. On our side also the application would make us always updated because medicine is always evolving. We would be able to know innovations happening in dentistry, how they manage this or that case, we would be lifelong learning. This would be helpful either for us or for our clients.

Interviewer: Do you think that this application can reduce the time you used to spend with patients? What can you tell us about that?

Interviewee: The time we used to spend while treating a patient? That is also possible because you can refer them to the application, giving them a link and telling them where to get it from, instead of spending a lot of time trying to explain to them. The time would be reduced a lot.

Interviewer: Yes. Now, which advices can you give so that all the materials and equipment needed in teeth scaling and polishing are useful for you? In case everything is ok for you, you can also tell us.

Interviewee: These equipment and materials should be in enough quantity because many patients are having a lot of calculus. Sometimes even when they have another chief complaint, you might wish first to clean their teeth before proceeding with the filling or the extraction, in order to work in a safe oral environment. We would also like to get the polishing paste whenever we need to use it. People start to be aware and to look for a better oral hygiene.

Interviewer: Towards the end of this interview, the last question which is similar to the one I asked before. Which advices can you give in order to make your job easier?

Interviewee: Based on my job experience, what I can advise is that our managers or you at the school of dentistry would plan trainings in order to refresh our knowledge on what is new in

the world of dentistry. Trainings would be very useful and would encourage us to keep enjoying our job.

Interviewer: In which domains would you wish to be trained?

Interviewee: Based on our daily activities, we would like to be trained in tooth restoration, disimpaction, bleeding management, drug prescriptions and use of antibiotics.

Interviewer: Thank you. The information you gave us is very important. We really appreciate.

Interviewee: Yes.
