# Supplementary material for: Evaluation of oral health services and challenges faced by oral health practitioners working in Nyarugenge, Rwanda
Source: PLoS One. 2024 Aug 19;19(8):e0309127. doi: 10.1371/journal.pone.0309127 (PMC11332939; doi:10.1371/journal.pone.0309127)
Supplement: S1 Dataset — (ZIP) [file pone.0309127.s001.zip › dataset/Dataset qualitative interview transcript/PARTICIPANT (14).pdf]

INTERVIEW WITH PARTICIPANT 14

Interviewer: Thank you for accepting that we have this interview

Interviewee: Thank you too

Interviewer: We are conducting a research about the importance of an application which would be put into the phone, in educating patients about oral health. We would like that you answer freely because every information will be kept confidential and there is no wrong answer, every answer is important. We are requesting your permission to record your answers so that we don't lose anything of what you will tell us.

Interviewee: No problem, you can record the voices.

Interviewer: Thank you very much. Now tell us, how do you feel about your work?

Interviewee: Maybe I can start by presenting myself

Interviewer: (interjecting) It is not necessary to tell us your name, it is not allowed in a research

Interviewee: Ok. Thank you. The work is good, many patients are coming to us and we help them as we can. They return home satisfied.

Interviewer: We would like that you tell us briefly how you feel about your work currently. If your job is tiresome, if you are pleased to do that job, if sometimes you have to rash and work very quickly in order to clear the line, if there are some challenges, feel free and tell us about how it is.

Interviewee: Thank you. Concerning our job, if you don't like it, it would be very challenging but when you like it, you enjoy doing it and the challenges appear small because even though the patients are many, you try to help them in a quick way concerning their chief complaints. Sometimes you can even give them an appointment in order to treat other problems you discovered in their mouth, which they were not aware of. However, we cannot overlook the challenge linked to the great number of patients even though this cannot be an excuse of not caring for them and helping them. We don't send anyone back home without treating them.

Interviewer: It means that for you the job is not so tiresome because you enjoy doing it.

Interviewee: Yes. Since we enjoy our work, it is not tiresome.

Interviewer: Were you expecting to receive such a great number of patients on a daily basis?

Interviewee: Anyway, since I stay here in town, I was expecting this but maybe at a lesser level compared to the current reality. We live in the town which has many inhabitants and people who need dental services are many, despite that there are still many who are not aware that they need dental treatment. I expected that patients would be many but the number is much bigger than what I expected to receive.

Interviewer: It means that for you, you were psychologically prepared, nothing surprised you

Interviewee: Yes, I was prepared

Interviewer: Very good. Now, tell us about giving oral health education to all patients who come to you. Tell us, is it really possible? If it is not possible or if it is very challenging, can you explain to us why?

Interviewee: Giving oral health education to all patients is difficult but it is possible. We do it but honestly, it is not all the time. Since there is a lot of work, we do it times to times and even then, we do a mass education. It means that sometimes we go to the reception having didactic materials and teach them like for ten minutes about oral health. Another way in which we do oral health education is during treatment. We treat while interacting with patients, telling them how they can brush their teeth and that they should visit dentists twice a year. We do our best even though the workload is heavy, we don't treat them silently, we talk to them about oral health.

Interviewer: Thank you. What you are doing is good, it shows that you value human beings, that they are not robots. You told us that it is possible but not always. What are the challenges that prevent you to do it every day?

Interviewee: Well, challenges that prevent us to do it every day are linked to the big number of patients. When you arrive and see that there are many waiting for you, you understand that you cannot get the time for teaching. You must start receiving them immediately in order to be able to help them all.

Interviewer: Yes. You told us that sometimes you go at the reception and give oral health education to all the patients. What are the main topics that you tell them about?

Interviewee: First of all, we remind them that everyone has teeth inside his/her mouth and that those teeth are also body organs like the others. We also tell them that teeth must be cared for and be cleaned. We teach them how to do oral hygiene, that they should brush their teeth in the morning, and at night before going to bed. We also show them the proper technique they should use while brushing their teeth. We teach them that they should change their toothbrush every three months without waiting that they are visibly spoiled. We tell them also that they should visit dentists every six months. We also tell them that when their gums are bleeding, they should go to the dental practitioner for help because we realized that many neglect that. Another thing we tell them is that when they observe many substances which attach to their teeth surfaces which the brush cannot remove, they should approach the dental practitioner for help. Briefly, anytime they observe something unusual on their teeth, they should go for dental check-up and for any eventual treatment.

Interviewer: When you are giving that oral health education, what didactic materials do you use in order to show the best way of brushing teeth?

Interviewee: First of all, we have didactic materials in our office. We have a big toothbrush and a big model that we use to show them the technique for tooth brushing. We also show them the gum and the tongue on the model. You see that this model is showing all the parts of the mouth.

Interviewer: Do you sometimes show to the patients the interdental floss? Do you also have it?

Interviewee: We have not yet started to show it to them but we tell them about it. We give them that information that the dental floss exists but we don't show it to them.

Interviewer: Thank you so much. Now, tell us about scaling of teeth. Is it possible that you provide that treatment to every patient who need it? Are there many patients who need it? How is it? Tell us about it.

Interviewee: Well, we cannot be able to do scaling for every patient who came to us. However, there are some patients who come looking specifically for that treatment. In that case, we do teeth scaling for them. For the others, when we see that they need teeth scaling, we tell them and give them an appointment for scaling. It means that for professional cleaning, we give an individual appointment. Since we have so many patients, we cannot do scaling for all the patients the same day. We give them different appointments. Even the one who is not aware

that teeth can be cleaned professionally, we tell him/her and after giving that information, an appointment is fixed for scaling.

Interviewer: Thank you so much. It means that the only challenge you have that hinder you from cleaning teeth for every patient who needs it is because patients are so many.

Interviewee: Yes, patients are many but even then we give them an appointment, asking them to come early in the morning and they try to respect the appointment and come. But, we cannot provide that treatment to all the patients who need it on the same day because they are many and they need other types of dental treatment.

Interviewer: Yes, we understand. The good thing is that you plan and offer them that treatment at a proper time. Tell us now about the sterilization of the scaling instruments. How is it?

Interviewee: Concerning sterilization of instruments, we never lack instruments here. We are privileged because this health center belongs to the catholic church so that basic instruments are there. Even though they cannot be enough for all the patients but we clean and sterilize them. Our instruments are safe because we have a sterilizer in which instruments are ready in 45 minutes. We don't have any problem about that.

Interviewer: It means that you have all the required instruments for teeth cleaning i.e. scaling and polishing scaled surfaces?

Interviewee: Yes, we have them. Whatever we need for scaling and polishing we have them.

Interviewer: It cannot happen that you send a patient back home because there are no sterilized instruments?

Interviewee: No, it cannot. Instruments are there.

Interviewer: Let us now consider that you have just finished to treat a patient. Maybe you have provided a mass oral health education at the reception. How do you give oral health instructions related to the treatment offered to them?

Interviewee: When I have just finished to treat a patient, I give them instructions related to what I did for them. As an example if I have just extracted a tooth, I put a gauze or a piece of cotton over the socket and ask them to remove it after thirty to forty minutes. After removing it, I tell them that they should avoid disturbing the socket with fingers or with the tongue

Interviewer: It means that you are able to find time to give those instructions to every patient you treat according to what you did for them?

Interviewee: Yes, I do because if you don't give them post-operative instructions, there might be some complications.

Interviewer: You told me that here, you have all the needed dental instruments and materials. What about the quality of care that you provide? How is it?

Interviewee: I cannot say that the quality is very high or very low, I can say that it is an average. The reason why I say that is because we have a big number of patients who come to us. The ideal time that I should spend with a patient might be shortened because the line is big. That is so disturbing for us because if it was possible to treat fewer patients per day, the quality of care would be superior because the patients would be more relaxed and I would interact with them more and provide every treatment they need. But since patients are so many, this will cause him to come many times because we cannot do different treatments in a single visit.

Interviewer: Thank you so much. Tell us now about equipment. You told me that you don't have any problem about instruments; but how about equipment? By equipment we mean like the dental chair, the sterilizer, and others; tell us about them.

Interviewee: Concerning equipment, since we have many patients who need our services, it would be much better if we had more equipment (door opening). Currently we have only one dental chair, it would be better if we had two of them. More equipment should be purchased in order to serve the population better.

Interviewer: Let us now talk about the available equipment. When one of them gets spoiled or is not functioning well, does the administration hurry up to repair it? Do they replace the spoiled piece without delay? How is it?

Interviewee: Anyway, there is a particularity of this health center. When I say that we need a certain equipment or instrument, it is almost automatic to get it because we do a requisition, and after like 45 minutes, the supplier brings it to us because we are in town, it is not challenging for us to buy instruments and equipment. What I say is that when we call the suppliers of dental materials, they immediately bring them and we pay them.

Interviewer: Even when it is one of the expensive equipment?

Interviewee: There is no problem here even for equipment. I can say that our administration is valuing dental service even more than I do.

Interviewer: Thank you so much. We have said that after doing a tooth scaling you must also polish that surface, you know it.

Interviewee: Yes

Interviewer: What about the polishing paste. Is it always available? Don't you sometimes have a stock out of it?

Interviewee: We can miss it but on our side, we never allow the stock out to happen. We always have reserve materials in our store.

Interviewer: Really? It means that you never miss the polishing paste, you always have it.

Interviewee: We never miss it unless it is nowhere to be found in Kigali.

Interviewer: But you told me that you never have a stock out. All your instruments and materials are always available.

Interviewee: We never have a stock out situation because here at our health center, we like dental.

Interviewer: Thank you so much. We are really happy that dental is valued.

Interviewee: For sure, here dental is valued a lot.

Interviewer: Let us now talk about how you feel about your security while treating a patient.

Interviewee: When I am treating a patient here, my security is assured. First of all, I am a hundred per cent confident about what I am going to do because I know how to do it, and

Interviewer: (interjecting) and what about fearing that you might contract a disease while treating? How secure are you on that side?

Interviewee: I cannot say that I don't think about that, I am a human being. That is why I must put on all personal protective equipment which help me to protect myself. I wear a face mask, a face shield, I really do my best to protect me. When hazard would happen like injuring myself with a needle after giving anesthesia, at that time, here we have a service which can help both

my patient and myself and do a test to check if any of us might have an infectious disease. It has never happened to find that the person was infected but in case it was discovered that one of us is infected, they would help us very quickly. However, I try to protect myself and to protect others.

Interviewer: It means that all personal protective equipment is available, no problem on this side?

Interviewee: Yes, they are available.

Interviewer: Now, what would ease your work in general?

Interviewee: Well, first of all what could ease my work is to be able to treat reasonable number of patients per day. For that to be possible, another dental staff should be recruited so that dental practitioner/ patients ratio might be appropriate. That goes hand in hand with dental equipment so that I might help my patients.

Interviewer: It means that you were working alone until now?

Interviewee: I was working alone but as they realized that the line of patients is becoming longer and longer, taking me like almost twelve hours every day, bending my back, treating and talking from seven am to five pm, at the same time making patients' records, they realized that it was really impossible to care for them alone and they recruited another dental therapist, currently we are two.

Interviewer: What impact that had on your working routine?

Interviewee: That had a good impact because when one is doing intraoral examination, another one is treating. If one is entering patient's data in the machine, the other one would be treating and when he/she is tired, we exchange our roles.

Interviewer: It means that if they brought more dental chairs you would have space for them?

Interviewee: Yes, there is a Rwandan proverb which says that 'no one can get a child and miss how to raise him', if we get that chair we can manage to secure a space for it.

Interviewer: Thank you so much. Now, if there was an application which would be installed in patients' telephones in order to give oral health education in general, what importance that would have for you?

Interviewee: If that application could be available, the importance would be very high. Sometimes people delay on phones looking at rubbish things, but if that application was installed there, it would help us to educate our patients. Sometimes we don't educate all the patients due to the heavy workload or the long line of patients, but if that application was there, the patient or the client would check it and we would share information about oral diseases and how to prevent them, oral hygiene and other topics through it. You understand that this application would be where we are not.

Interviewer: Do you think that this application can reduce the time you used to spend with patients teaching them? What can you tell us about that?

Interviewee: It can reduce it a lot. Let us say that because of the application the patient has already seen the best technique for tooth brushing, they already know how to use the dental floss, they already know how to take care of deciduous teeth, they have already watched videos in the application; you see that the interaction would be much easier because they would respond yes, I already know about what you are telling me. On the other side, if they have never seen that application, they keep asking you to show them how to do it. If they watch the application, I can show them as a reinforcement but it cannot take a long time. That application would be very helpful for us.

Interviewer: It means that for you, the application is very important and it can simplify your work.

Interviewee: Yes

Interviewer: Thank you so much. Now, which advices can you give so that all the materials and equipment needed in teeth scaling and polishing are useful for you?

Interviewee: To who this advice should be addressed?

Interviewer: You would give this advice to whoever is concerned like employers, or dental health policy makers.

Interviewee: From our health center view, the advice that I can give is that, starting from the higher governmental levels who must overlook for the population health, they should also think much about oral health. Yes, it is clear that they consider health in general but they should not neglect oral health because it is very important. If they do, health facilities would receive dental equipment and materials more easily, which would allow them to care for oral health of the

community in general. These should be availed but at the same time the number of dental practitioners should be increased in order to satisfy people who come to us. If there is enough dental staff, even when patients are many, we would manage to teach them because one would be doing oral health education while the other would already start treating patients. In that way, people would be aware that they need dental check-up even when they are not sick because currently people come only when they are sick. If we have time to teach them, they would take that initiative to come for check-up even when they don't have any problem.

Interviewer: Thank you so much. Those advices have already answered the following question but if you think that you can add something on it, go ahead. The question goes like this: Which advices can you give in order to make your job easier?

Interviewee: Well, for my job to be easier, I must be punctual and arrive on time at my place of work meaning that my transportation means should be made easy. Another advice is that when I get to the place of work, the instruments and materials should be readily available so that I don't miss anything to use in the middle of performing an act because this would be very disturbing. Equipment, instruments and materials should be there and well prepared, ready to be used.

Interviewer: You say that they should be well prepared, that is important because if they were there but not well prepared, meaning if they are not sharp, if they are not effective, they would be useless. I think this is what you wanted to tell us.

Interviewee: Yes.

Interviewer: Nothing more you can tell us that would ease your work?

Interviewee: Another thing, today we are two dental therapists but we have a really big number of patients, like seven hundred a month, reason why we would need another dental practitioner in order to work as a team.

Interviewer: Thank you so much. The information you gave us is very important and it will help us in this research we are doing. It will also be useful for the general dental health. Thank you.

Interviewee: Thank you too.
