# Supplementary material for: Evaluation of oral health services and challenges faced by oral health practitioners working in Nyarugenge, Rwanda
Source: PLoS One. 2024 Aug 19;19(8):e0309127. doi: 10.1371/journal.pone.0309127 (PMC11332939; doi:10.1371/journal.pone.0309127)
Supplement: S1 Dataset — (ZIP) [file pone.0309127.s001.zip › dataset/Dataset qualitative interview transcript/PARTICIPANT (2).pdf]

INTERVIEW WITH PARTICIPANT 2

Interviewer: Thank you for accepting that we have this interview. We shall try not to take a lot of time. As we told you, we are doing a research related to the PhD of our colleague. He would like to know the challenges that dental practitioners are meeting in their practices and the role of an application which would be installed in people's telephones in order to give oral health education. We would like to know if it would be useful. As we told you, the research is confidential and there is no wrong answer, all answers are correct and important. It is for that reason that we would like to record the voices so that we don't lose any information. Do you give us that permission?

Interviewee: Yes

Interviewer: Let us start. We would like that you tell us briefly how you feel about your work currently. If your job is tiresome, if you are pleased to do that job, if sometimes you have to rash and work very quickly in order to clear the line, if there are some challenges, feel free and tell us about how it is.

Interviewee: I started the work here two years ago. There are not many challenges in my work except the lack of dental materials, even though this is a referral hospital. Otherwise there is no problem about the way the work is planned, the number of dental staff, the team spirit displayed, and the number of patients.

Interviewer: It means that, apart from the lack of dental materials, you are happy with your work?

Interviewee: Very happy. The only problem is shortage of dental materials

Interviewer: It means that here you are never overloaded by the number of patients?

Interviewee: We are lucky because many times we have university students. If they were not there, it would be challenging but when they are there, they help us so much.

Interviewer: Now, when you were still studying, were you expecting to receive many patients or only few? How was it?

Interviewee: I didn't have any specific expectations because I didn't know where I would be working, especially that Public clinics are different from private ones. I made my mind to adapt to any situation I would find on the field.

Interviewer: Now, tell us about giving oral health education to all patients who come to you. Tell us, is it really possible? If it is not possible, can you explain to us why?

Interviewee: It is not possible. Even when they come on appointment, the patients we treat don't know why they came and what we would do for them. I don't know if it is our fault or if it is due to lack of time but what I can say is that oral health education for patients is not done.

Interviewer: You really don't know the reason why? I think you know challenges hindering you from doing it.

Interviewee: Even though we don't have many patients, seating the patient on the dental chair and spending like twenty minutes educating him/her while there are other patients behind them can be time consuming.

Interviewer: It means that the challenge you meet is time

Interviewee: Yah, of course

Interviewer: When you manage to give oral health education to some patients, which are the main topics do you tell them about?

Interviewee: Oral hygiene. That is the basic of that we may tell them.

Interviewer: What do you mean by oral hygiene?

Interviewee: We tell them about brushing, that is it.

Interviewer: Do you tell them only in theory or you also demonstrate for them?

Interviewee: No, no. We only tell them to remember to brush their teeth in the morning and at night before going to bed. Only that.

Interviewer: But don't you show them the technique?

Interviewee: No

Interviewer: Why aren't you able to show them that?

Interviewee: First of all, it is because of time. Secondly it may be linked to our routine of work. We only remember to tell them about brushing but we have not yet realized that we should even show them how to do it.

Interviewer: If you were willing to do it and in case you had time for doing it, do you have didactic materials which would help you in the demonstration?

Interviewee: No, so far we don't have.

Interviewer: You don't have. Now, tell us about scaling of teeth. Is it possible that you provide that treatment to every patient who need it?

Interviewee: No, it is not possible here because this is a referral hospital where patients come from other hospitals, having dental problems which were not addressed in these hospitals. So, in case a patient had an accident and we put intermaxillary fixation, we can do scaling for him/her after removing the wires but for a patient who came for an extraction, sometimes we tell them to go back to the referring hospital for scaling because we don't have time for doing scaling for all the patients who come here.

Interviewer: It means that everything is evolving around time. Are there many patients who need it?

Interviewee: Scaling? If I estimate, 80% of all the patients we receive need dental scaling.

Interviewer: I would like to ask you about the instruments for scaling and polishing dental surfaces, do you have enough of them? If it was necessary to do it, how many patients could you treat per day?

Interviewee: Usually we schedule two patients per day for scaling. The biggest number we can treat is four patients per day. Currently we only have two ultrasonic scaler tips meaning that we serve a patient and another has to wait to be treated after the sterilization. If it was necessary to do many dental scaling, we could not find instruments.

Interviewer: Tell us now about the sterilization of instruments. How is it?

Interviewee: There is an improvement these days. It is good.

Interviewer: Expand a little more on that

Interviewee: Before, we used to send instruments to the general sterilization department of the hospital but now we bought new sterilizers which are available here in the dental service. We do the sterilization ourselves and we also have a trained staff to do it. There is a real improvement.

Interviewer: Since when do you have these new sterilizers?

Interviewee: Since the beginning of this year. That is when the first new sterilizer was received.

Interviewer: You were about to say that it is small. Is it really small? Meaning that it is not enough for you?

Interviewee: (both smiling). Yes, it is small compared to the others However, it is sufficient for our service. When it becomes necessary, we send instruments to the general sterilization department of the hospital.

Interviewer: Does it sometimes happen that you send a patient back home because there are no sterilized instruments?

Interviewee: Yes, it happens

Interviewer: Really? Especially for what types of patients?

Interviewee: Like patients who need disimpaction or any other patient who cannot wait. A patient who needed scaling may say that it is taking too long to sterilize the instrument and then decide to go.

Interviewer: You told me that it is somehow impossible to give oral health education before the treatment. What about giving post-treatment instructions? Do you do it? Is it possible for you?

Interviewee: Of course we give post-treatments instructions but we don't go into details. Maybe one practitioner can remind the patient to clean the teeth but not in deep.

Interviewer: You only give instructions related to what you did to the patient?

Interviewee: Yes. If we removed the tooth, our instructions will be related to that. We don't talk about routine oral healthcare.

Interviewer: You have been here for two years now. What can you tell us about the quality of care that you provide? Are you happy with it? Can it be improved in any way? How is it?

Interviewee: Compared to how it was at my arrival, things are slowly improving. More dental staff are being recruited, dental materials are availed, and the services provided are increasing. You have seen that today they launched the service of dental laboratory. Before, we used to send the patients in private laboratories for dental prostheses. However, still much need to be done but slowly by slowly it will be better.

Interviewer: That is really good news if you are stepping ahead. According to you, what could be added in order to improve more the quality of care?

Interviewee: (hesitating). Like now that I am affected in maxillofacial department, I think they should train more specialists. Currently we have only one maxillofacial surgeon dealing with accidents and emergencies. You find that a patient with orofacial cancer can die without even being examined by the specialist. The doctor does consultations only once a week on Thursdays and performs surgeries on Fridays. You understand that only one or two patients can be attended on a weekly basis while he has almost one hundred patients on the waiting list. If you would come here on Thursdays or go to Rwanda Military Hospital, you would see it by yourself, the number of patients looking for that maxillofacial surgeon is big. That is a big gap. Another thing, skills we get from school are really not enough. Of course the university tries its best so that we get some exposure but when you come to the field, you realize that there was more we wish we could have learnt. I base my observation on the dental surgeon interns we receive here. They can spend a whole year before we trust them about a given procedure. That is why I say that academic training should be improved.

Interviewer: Thank you so much. Tell us about equipment; by equipment we mean like the dental chair, the sterilizer, the compressor and others. If one of them get damaged, what happens?

Interviewee: That is what I was telling you before, that there is a tangible improvement. We have a local technician who is permanently here at Hospital. Before we had to wait the supplier like KIPHARMA but no more. When any equipment has a problem, if the dental chair crushes for example, we call him and he immediately comes, unless when he finds that it is beyond his

competences. In that case, he waits for the suppliers, when the guaranty is still valid, who come and repair it.

Interviewer: What equipment is causing him more struggle to repair?

Interviewee: The dental chair. They train him but he is still not mastering that.

Interviewer: He doesn't feel very confident. What about the sterilizer, does he also repair it?

Interviewee: Yes, he does. Yesterday he was here repairing it.

Interviewer: Here when you do scaling, do you also do polishing?

Interviewee: No, unless there is a patient who came complaining of stains. Otherwise, we never do polishing. Due to time, we only remove calculus and tell them to come back after six months, especially that currently we don't even have polishing paste.

Interviewer: You don't have polishing paste?

Interviewee: That is what I was telling you concerning stock out of some materials.

Interviewer: How secure do you feel when you are treating patients, especially on the side of the risk of contracting an infectious disease?

Interviewee: We try to protect ourselves with PPE. We have face shields, medical coats, gloves, and all of that, except when the practitioner him/herself neglects to do it. Only we don't have surgical gloves, they are used only in theater. We also don't use head covers.

Interviewer: Now, what do you think would ease your work in general?

Interviewee: Since we receive patients for which it was not possible to address their cases in district hospitals, it would be good if materials were availed. That is what I think. The most challenge is linked to dental materials.

Interviewer: Dental materials. What do you mean by materials? Are you talking about consumables? Equipment?

Interviewee: Either consumables or equipment. Both are still a challenge.

Interviewer: Now, if there was an application which would be installed in patients' smart phones in order to give oral health education in general, what impact that would have on your daily work?

Interviewee: That would be very helpful to us. There are some cases that we receive and we realize that the reason why the patients came so late is because they didn't have information. If they were aware before, they would have come for help and the problem would have not reached that stage. That is where the application would be very helpful to us.

Interviewer: In which other areas that application would be useful?

Interviewee: The application would be also useful in the community especially for mothers and children. If the population was informed, people would perform some acts of prevention like dental cleaning for children, because they still complain about the price of dental services.

Interviewer: You kept telling me about the challenge of time, time, time. Do you think that this application can reduce the time you used to spend with patients? What can you tell us about that?

Interviewee: Yes of course because even though we are rushing, there are some patients who refuse to leave without information, and we are obliged to teach them despite the big line of patients outside waiting for us. The application would help us in that area.

Interviewer: Yes. Now, which advices can you give so that all the materials and equipment needed in teeth scaling and polishing are useful for you? So that you might enjoy using them?

Interviewee: At the beginning of the year, the administration of the clinic always makes a requisition of needed materials for scaling and polishing. Since those are consumables who might finish and not get replaced immediately, they should consider that they are important and avail them. As an example, people don't seem to worry about the shortage of the polishing paste.

Interviewer: You told us that currently you only have two ultrasonic scaler tips. For which reasons there are only two?

Interviewee: I think that we practitioners are responsible for that because usually, the request is made and that items are delivered but after one month, you can find that only one is still functioning while others have been thrown in the dustbins.

Interviewer: It means that the advice you can give is that everyone should feel responsible and take care of the instruments and materials they use.

Interviewee: The same way we cannot keep working without a handpiece, they should think that the scaler tip is also important, that the work cannot continue without it.

Interviewer: Which advices can you give in order to make your job easier in general?

Interviewee: That is what I was talking about concerning the availability of dental materials.

Interviewer: Thank you so much. The information you gave us is very important and it will be helpful for us. Have a nice working day.

Interviewee: Thank you very much
