# Supplementary material for: Evaluation of oral health services and challenges faced by oral health practitioners working in Nyarugenge, Rwanda
Source: PLoS One. 2024 Aug 19;19(8):e0309127. doi: 10.1371/journal.pone.0309127 (PMC11332939; doi:10.1371/journal.pone.0309127)
Supplement: S1 Dataset — (ZIP) [file pone.0309127.s001.zip › dataset/Dataset qualitative interview transcript/PARTICIPANT (3).pdf]

INTERVIEW WITH PARTICIPANT 3

Interviewer: Thank you so much. As you see, we are about to have an interview with you related to the challenges you might be facing in your daily work and about the importance of an application which would be installed in telephones and which would be delivering oral health education. We shall try not to take you a long time because we are aware that you are the only one dental practitioner here, but feel free and give us all the information. There is no wrong answer, all the answers are important because they will be useful to us in this research. We would like to ask for permission to record the voices, if you accept, you tell us so that we continue.

Interviewee: You can record no problem

Interviewer: Thank you so much. Let us now start. How do you feel about your work currently?

Interviewee: Since we do this work every day, it is not very difficult except that there are a lot of patients especially on Mondays and Fridays, at the beginning and the end of the week.

Interviewer: Based on how it is, are you pleased to do that job? Is your job tiresome, do you sometimes have to rash and work very quickly in order to clear the line? Are there some challenges? Feel free and tell us about how it is.

Interviewee: Since the patients are many, sometimes I have to rash in order to serve all of them. Even now there were many, I examine like ten of them, put anesthesia to five at the same time and do teeth extraction for them. After I receive another five and so on. However, they are not so many today.

Interviewer: Do you enjoy that work? Is it very straining for you? How is it?

Interviewee: Since it is what I do every day, I enjoy it but it is tiresome.

Interviewer: Can you explain in which way it is tiresome?

Interviewee: It is tiresome because patients are many and you cannot satisfy all their needs. Some come for scaling and fillings, and because there are many patients, I am obliged to give them an appointment.

Interviewer: Were you expecting to receive such a great number of patients on a daily basis?

Interviewee: No, I was not expecting this especially that at the beginning, patients were not many. I used to receive like ten, five or eight patients a day. It was rare that I received up to fifteen patients on Mondays but currently, maybe because they are informed that dental services are available at the health center, they are so many.

Interviewer: You told me that you were not expecting this. How did you react when it happened?

Interviewee: At first, the transition was challenging for me because I was used to receive few patients but since that is why you applied for that job, you wish to receive enough patients so that the health facility you are serving might also have gain from your services.

Interviewer: (repeated noise from door opening and closing abruptly). (If you have the key can you close that door please?). **Tell us something about providing oral health education for every patient that you receive. If you find it possible to do it for every patient or if it is challenging, tell us how it is.**

Interviewee: Giving oral health education to every patient is challenging because patients are many. You only tell them about the act itself you are about to do for them or after completing the procedure, giving them related instructions. Otherwise it is very challenging unless you do mass education but they don't come at the same time.

Interviewer: That means that you don't have time to give oral health education to your patients?

Interviewee: No time for it

Interviewer: You never do it

Interviewee: Yes

Interviewer: Since you tell me that you never do it, I cannot ask you how you do it and what you use.

Interviewee: For the patient I just treated, I teach him/her. As an example, if I have just done teeth scaling for a patient, I teach him/her how they should keep brushing their teeth.

Interviewer: That question was coming later but now we were asking you about general oral health education.

Interviewee: We don't do it

Interviewer: What about doing scaling of teeth for every patient who needs that treatment, is it possible for you?

Interviewee: Yes, I do teeth scaling for every patient who needs it but on appointment. I don't do it the same day they come unless it is on Wednesday, Tuesday or Thursday because clients are not so many on those days.

Interviewer: Are there many who need that treatment?

Interviewee: Almost all of them need that treatment.

Interviewer: And you are not able to respond to their needs

Interviewee: Since they have other dental problems for which they came, like dental pain, they consider that to be a priority and that is what I address first and then I give them an appointment for cleaning. However, mostly they don't respect that or they wait like two or three months before coming. When they come I do scaling for them.

Interviewer: What are the challenges you face which hinder you from doing it as it should?

Interviewee: It is only time

Interviewer: Can you expand more on that?

Interviewee: Since patients are many and tooth cleaning can take at least forty minutes depending on the intraoral situation, I cannot get that time and be able to receive other patients.

Interviewer: Tell us now about the sterilization of instruments.

Interviewee: I use all the available instruments and clean them at the evening. I sterilize them in the morning when I arrive at work.

Interviewer: Let us consider like those days when you receive patients for dental scaling, how many patients can you treat based on the available instruments? Because you tell me that you use instruments you have, clean them in the evening and sterilize them the following morning.

Interviewee: I have instruments that can serve like five patients who need dental scaling

Interviewer: Don't you ever miss instruments for tooth scaling when you wanted to do it?

Interviewee: Since I examine them before, I first check the number of instruments I have and when I see that they are enough, I keep examining patients until the number is reached. Then, for the remaining patients, I give them an appointment for the following morning.

Interviewer: Apart from scaling, in general, you told me that you examine the patients based on the available instruments, how many patients can you treat per day?

Interviewee: I can treat up to thirty patients per day.

Interviewer: Offering different kinds of treatment?

Interviewee: Yes

Interviewer: Why cannot you sterilize the instruments in between so that you keep using them as they are needed?

Interviewee: Since I am the only staff in the dental service, doing all the work myself even that cleaning of instruments and the sterilization, I cannot mix all of that. Another challenge is that to be fully sterilized, instruments spend like two hours in our sterilizer. You understand that if I go for sterilization at 02:00pm, they would be ready at 04:00pm and it is becoming late to start receiving more patients.

Interviewer: It means that the sterilizer is not available here in the dental service, you go elsewhere for sterilization?

Interviewee: Yes, I do it from a common sterilization environment for the whole health center.

Interviewer: What are the reasons why you are doing all alone? No one is in charge for sterilization?

Interviewee: That is the habit of the house which I found here because the health center is led by catholic sisters. Whoever has used instruments and materials in a given service is also charged to clean them, sterilize them and range them at their appropriate place.

Interviewer: Since you told me that you examine the number of patients based on the available instruments, it means that sometimes you fail to offer treatments because the sterilized instruments have been all used?

Interviewee: Yes, that sometimes happens when patients are many and instruments are few. They return home and come back the following day.

Interviewer: You told me that after treating a patient, you take enough time to explain to him/her what you have done. Is that true?

Interviewee: Yes, I take time to explain what I did for them. If for example I did a tooth extraction for them, I give instructions on how to behave after that extraction so that the healing process might be good. If I did teeth scaling for them, I give instructions on how to maintain a good oral hygiene. If it is a dental filling that I have done, I also give post-operative instructions based on the type of filling I have done so that they protect it.

Interviewer: Is there any challenge you meet during that process of giving post-treatment instructions?

Interviewee: Since these instructions are very necessary, there are no challenges, I must give them in any way.

Interviewer: When you consider in general, what can you tell us about the quality of care provided here?

Interviewee: Thinking about the quality of care offered to them, the tariff of dental acts offered here at the health center is not clear. That makes that sometimes I cannot offer a service that the patient needs and which I am able to perform, simply because the community based health insurance doesn't cover it. In that case, I cannot offer that service and I refer thepatient. You understand that the patient will not be happy when that happens. For these reasons, some patients are not satisfied with our service, because they don't receive what they needed due to the insurance coverage.

Interviewer: And how do you feel in such instances?

Interviewee: When the patient leaves you unhappy, you cannot be happy either because you realize that you didn't offer the service as it should. Even though you were not responsible for that situation, you feel sad. On the other side, when you have provided a service to a patient and they don't believe that what you did was appropriate, you try to teach them but if they don't understand you refer them to higher level health facilities for more enquiries about the service offered.

Interviewer: Does it happen very often?

Interviewee: Yes, it happens. Sometimes you do an extraction of a tooth and afterwards the patient comes back saying that parts of the tooth are still there while what they feel is the alveolar bone in which the tooth was anchored. When they are not convinced, we refer them.

Interviewer: Tell me now about the equipment like the dental chair, the compressor and others. When one of them gets spoiled what happens? How does the administration react?

Interviewee: When something gets spoiled, the administration understands and looks how to repair it and even when something needs to be bought, I tell them and they buy it. Maybe not immediately on that day but the buy it, they don't complicate us.

Interviewer: They never neglect or delay after that you inform them about any spoiled equipment?

Interviewee: When I tell them and realize that they don't respond to it quickly, mainly because they have a lot of work, I return and remind them. After that, they understand and tell me to do a requisition, to look for proforma invoices from two suppliers and after that they pay them and bring them to me.

Interviewer: It means that you don't have any problem concerning equipment, instruments and materials.

Interviewee: I cannot have such a problem. I make a judgement and when I see that some items are not very needed based on my clients and I don't request a lot of them so that the health center doesn't loose money.

Interviewer: Still on that aspect of scaling and polishing dental surfaces, you have all the required instruments and materials?

Interviewee: Yes, I have them

Interviewer: Sure? Even the polishing paste?

Interviewee: Yes, I also have it

Interviewer: Kindly expand a little more on that.

Interviewee: In relation to dental scaling and polishing, I really have the required items. When a patient feels that the scaled surface is not smooth, I polish it because I have the polishing paste and all the handpieces are well functioning.

Interviewer: Now, tell us about your security while treating patients. Do you feel secure? Aren't you afraid of anything?

Interviewee: On my side, I don't have any problem but sometimes I receive a patient with a case which should be managed by someone else or for which I should be assisted by someone else. The good thing is that I learnt and know how to manage that case but it can be challenging for someone else of my domain.

Interviewer: Can you give us some examples?

Interviewee: A person can be in shock and it becomes necessary for me to do an intravenous infusion and other needed care. That usually would require the intervention of a nurse but I do it myself without any problem.

Interviewer: It means that if you were lucky to get a nurse in your dental service, this would give you more security?

Interviewee: Yes, I would feel more secure.

Interviewer: That is about patient's security; what about your own security when you are treating patients?

Interviewee: My security is assured because no one can enter this room and aggress me

Interviewer: You are never afraid of being contaminated by the patient you are treating?

Interviewee: No, because I have all the necessary protective equipment. So, if I am contaminated, this would be due to my own error.

Interviewer: It means that you have all the personal protective equipment?

Interviewee: Yes, If I want I can get them all. Even face shields, I can use it if I need to.

Interviewer: You say that if you want you can get them. Does that mean that you don't want them so far?

Interviewee: No, when I am doing dental scaling I use them. I am wearing the face shield and my chest is also protected. Only when I see that it is not necessary I don't wear them.

Interviewer: What about eye protection? Do you have equipment for that?

Interviewee: Since I am wearing a face shield, my eyes are also protected

Interviewer: According to you, what can make your work much easier?

Interviewee: If they would recruit more dental staff, this would ease my work. If we are two, when I would be working on the dental chair, the other one would be receiving the patient, examining him/her in order to know what we have to do for them.

Interviewer: Is that all you need? If you get another staff, everything would be ok for you?

Interviewee: Another thing is to increase the number of instruments especially the ones needed during teeth extraction because they are few compared to the number of patients. If they are availed, my work would be easier.

Interviewer: Now, if there was an application which would be installed in patients' telephones in order to give oral health education in general, how to care for their teeth, what importance that would have for you?

Interviewee: This would help us a lot because it would reduce the time you should spend with the patient educating them on how to brush their teeth since the application would have already done it. So, if the patient comes for a certain procedure, they would not delay asking how they should brush their teeth or how to perform oral hygiene in general. The time you would take after the procedure to explain all of these, the application would have already explained.

Interviewer: You mean that this can shorten the time you used to spend with the patient?

Interviewee: Yes, it can assist you because sometimes the patient might come and ask you about how to clean his teeth and you take time to explain to them but if they have that application, they would not even ask you such question, meaning that the time spent with them would be reduced.

Interviewer: You told us that you never get time to do oral health education to patients. What would be the contribution of that application in that matter?

Interviewee: Since we don't have time for that, in case the application is there, it would educate the patients and all the patients would know about how to do oral hygiene because usually, it is only few patients who express that curiosity and ask you about it. We would get more time for receiving more patients.

Interviewer: Which advices can you give so that all the materials and equipment needed in teeth scaling and polishing are useful for you? So that they are more effective and so that you enjoy using them?

Interviewee: The advice I can give is that the act should be covered in the insurance scheme, especially the polishing procedure. If you use the polishing instruments and paste knowing that it will be reimbursed, you would be happier instead of using them knowing that it is a waste of materials.

Interviewer: How does is work currently?

Interviewee: Until now when you do polishing, it is not considered. They only recognize and cover only the scaling but when you see that the patient needs polishing and that you do it, it is not covered by the insurance.

Interviewer: It means that there are some patients for whom you do only scaling and not polishing?

Interviewee: I do it because I have the materials but it is not paid for.

Interviewer: I was asking that because you told me that you do polishing when you see that it is necessary. Do you sometimes see that it is not necessary to do polishing after doing a dental scaling?

Interviewee: Yes, sometimes after scaling you realize that polishing is not needed depending on the intraoral situation.

Interviewer: What are the instruments for scaling that you have?

Interviewee: We have high speed ultrasonic scaler

Interviewer: What about manual scalers?

Interviewee: They are also there but I don't like using them

Interviewer: Which advices can you give in order to make your job easier?

Interviewee: Since dental problems have become so many, children grow having dental problems and the prevalence of dental problems have increased in adults. So, the advices I can give are that there should be dental personnel affected in the community for educating people about oral health, that would be very good. That is why the application you were telling me about would be much useful, because problems start at the community level, not at the health

facility. Meaning that, since oral problems can be prevented, and that the application can help them in that, it would be good if it was installed in people's phones and was used; it would remind them how to clean the mouth.

Interviewer: That is very important at the community level but let us now talk about here at your place of work, about the work you do on a daily basis. Which advices can you give in order to make your work easier?

Interviewee: The advice I can give in order to make my work easier is that patients should arrive here in the dental service earlier instead of delaying them at the reception. That would allow me to receive them and offer them the service they need so that they return home quickly.

Interviewer: Thank you so much, all the answers you gave me are very important and they will be useful to us. (she asked the note taker to ask any question he might have)

Note taker: I would like to ask you another question related to infection control materials. Do you have cover rolls? Like the ones they use to cover handpieces.

Interviewee: No, we don't have.

Interviewer: Thank you so much. It will be very useful.

Interviewee: Thank you too.
