# Supplementary material for: Evaluation of oral health services and challenges faced by oral health practitioners working in Nyarugenge, Rwanda
Source: PLoS One. 2024 Aug 19;19(8):e0309127. doi: 10.1371/journal.pone.0309127 (PMC11332939; doi:10.1371/journal.pone.0309127)
Supplement: S1 Dataset — (ZIP) [file pone.0309127.s001.zip › dataset/Dataset qualitative interview transcript/PARTICIPANT (4).pdf]

INTERVIEW WITH PARTICIPANT 4

Interviewer: As we were telling you, we are conducting a PhD research about the challenges dental staff are meeting while treating Nyarugenge population but also the impact an application which would be put into the phone in educating patients about oral health would have on their work. In research there is no wrong answer, every answer is important. You also know that every information is kept with confidentiality, no one will know who said this or that. We would like that you answer freely and we are requesting your permission to record your answers.

Interviewee: No problem

Interviewer: Thank you so much. Let us start. We would like that you tell us briefly how you feel about your work currently. If your job is tiresome, if you are pleased to do that job, if sometimes you have to rash and work very quickly in order to clear the line, if there are some challenges, feel free and tell us about how it is.

Interviewee: The first challenge here is linked to the number of patients. Patients are many, they are not proportional to the number of dental clinicians. It is challenging to serve such a big number of patients while we don't have enough working stations, so that each one might work from his/her own dental chair. So, since we share the dental chair and that we receive many patients, it is challenging and it causes some patients to be given distant appointments. One might have come with an infection today but by the time to return on appointment, that infection might have become more severe despite the fact that we had given them antibiotics. You are creating the risk of antibiotic resistance while trying to help them. Again, a patient might come with a problem which was supposed to be addressed immediately but you put them the next day or on another day. That is always disturbing the schedules. Another challenge is linked to materials. Sometimes materials are not enough and when you receive many patients, materials should be also enough. Most of the time we don't have enough materials for all the patients. Some patients get a good service while the others get lower service because some materials have finished. You treat patients willing to help them but this help is not the same for all the patients. For some of them you try to remove the pain only, while waiting for more materials.

Interviewer: How do you feel when you face such a situation?

Interviewee: When that happens, you are not happy with the working environment. You wish you would go somewhere where all needed materials would be available so that you help people or you wish that whoever is concerned should try to make an advocacy so that we are facilitated to help patients as much as possible from here. Some materials are delaying due to hospital processes even if they are not expensive. The dental practitioner cannot take the initiative of buying materials to be used on patients. That is also a challenge. It doesn't mean that the materials are nowhere to be found, only the process is long. Sometimes you get them too late, when it is no longer possible to help the patients. That is so discouraging because you can realize that what you did to the patient was not perfect, not because you are not able, but because there is something missing, hindering you from offering a good service.

Interviewer: Were you expecting to receive such a great number of patients on a daily basis?

Interviewee: Here, I was expecting that because district hospitals don't have enough materials. As a referral hospital tries; what the other hospitals do is to refer many patients to us. Unfortunately, when they come, they find that even on our side, we are not well equipped. That is the challenge we meet. You understand that it starts from lower levels, at district hospitals and health centers.

Interviewer: Yes. Now, tell us about giving oral health education to all patients who come to you. Tell us, is it really possible?

Interviewee: Yes, but I do it especially for patients with chronic diseases like diabetic patients. Most of the time they come with dental problems. Usually they need a dental check-up twice or at least once a year but they wait to come when their disease has become uncontrolled and that their teeth are damaged. For such patients I take time to tell them how to perform oral hygiene because they have diabetes mellitus which goes hand in hand with dental diseases. I tell them how to brush their teeth and I remind them that it is necessary for them to come for check-up even when the teeth don't have any problem. For other patients it is also possible, they used to do it here. This was not done at individual level but since we work in wings, every wing had a day for mass oral health education for one hour in the morning. Sometimes even the nurse knew what to teach to patients and she did it before patients entered the consultation room. That helped patients to understand the importance of dental services and how to preserve a good oral hygiene. What I say is that it is possible to do oral health education in offices but

here it is challenging because of many patients. That is why you select who to teach according to the necessities, you don't give oral health education for each patient.

Interviewer: You told me that you used to give mass oral health education, what stopped you from continuing?

Interviewee: Usually oral health education sessions were planned. When we were still students, this was mandatory. I think that it is only by negligence because there is no any other reason. If the responsible of the clinic organizes that again and makes a duty roaster, this can be resumed again.

Interviewer: Now, tell us about scaling and polishing of teeth. Is it possible that you provide that treatment to every patient who needs it? Tell us about it.

Interviewee: All the wings don't have required equipment. Every patient who needs scaling get that treatment. The only challenge we have is linked to dental materials. Polishing tooth surfaces cannot be done because we don't have contra angle polishing brushes nor polishing cups. We have the material but the polishing brushes we have are not of good quality; they don't remove the stains well. If that was addressed, we would do it because our wing as well as the one for maxillofacial surgery are equipped for that. We can make a plan so that every patient who needs scaling and polishing receives that treatment.

Interviewer: Apart from the polishing brushes, do you have the polishing paste?

Interviewee: Yes, we have a lot of it.

Interviewer: And is it of good quality?

Interviewee: Yes, it is of good quality, like the one we used when we were still students. The polishing paste is ok.

Interviewer: You say that whoever needs dental scaling receives it?

Interviewee: Here in our wing, we do it for every patient who needs it or for which we see that the problem he/she has was caused by the lack of dental scaling, we do it for them. They cannot go without getting their teeth scaled.

Interviewer: Sure? Tell us now about the sterilization of the scaling instruments. How is it?

Interviewee: Sterilization of instruments?

Interviewer: Yes

Interviewee: On my side, there is no problem about the sterilization of instruments. But as I told you, the instruments are still a challenge. Some are many, others are missing while they were mandatory but for those we have, there is no problem about sterilizing them. We have a staff allocated to that and for me she/he is doing it well so that we don't miss instruments. After using the instruments, you hand them to him/her, they clean them and sterilize them. By the time you finish the treatment, others are ready for use in case you need them.

Interviewer: Really? It means that you have your own sterilizer here in the dental clinic?

Interviewee: We have two sterilizer machines. We used to have another one which was bigger but it got damaged but currently we have two. For big instruments, they carry them to be sterilized in the general sterilization department and bring them back in the morning before we start the treatments. Sterilization here is not a problem.

Interviewer: Sure? It means that you can never fail to treat a patient due to lack of a sterilized instrument?

Interviewee: Even if it is at seven or eight or even during the weekend, you can treat a patient. The sterilizer machines are there and most of us know how to use them, so that even if the cleaner is not around, you can do it yourself. Most of the times, sterile instruments are there in enough quantity.

Interviewer: You told us that you sometimes take time to give individual oral health education especially to patients with chronic diseases. What about discussing with the patient after treatment?

Interviewee: Telling you the truth we don't do it much, depending on the time you have or if it is a patient who is curious and asks questions. When it is a patient without curiosity, we give them an appointment and tell them what you plan to do and that is all. When it is a patient who is curious and asks questions, you cannot escape without explaining to him/her but it is challenging because we have many patients who need consultations and others who came on appointment for treatment. Furthermore, we share one dental chair and we are rushing to clear the line on time. When it is a patient who asks questions or the one who has a condition which necessitates oral health education, you do it briefly.

Interviewer: What about post-treatment instructions?

Interviewee: That we do but we prefer telling them everything before. We inform them about the whole treatment plan unless when something goes wrong in the middle of the treatment or if there is a risk of treatment failure. We start afresh and explain but at the beginning, for example for a patient who needs scaling, we explain what we shall do. Either scaling his/her teeth straight away or giving first sodium bicarbonate and a toothpaste before scaling in order to smoothen calculus. We can tell them that we will first give them sensodyne toothpaste to be used before scaling and few days after scaling. We explain all the procedure before and we welcome eventual questions and clarify them. After finishing the treatment, we don't repeat everything because they are already informed.

Interviewer: And you think that post-operative instructions are not necessary?

Interviewee: Instructions are mandatory because they are factors of the success of the treatment. If you don't give instructions after a root canal treatment, the patient can go and use the tooth while it is empty, packed only with the cotton inside and it can break. You tell the patient not to use that side until the time we will close it. Such instructions are given.

Interviewer: When you think about the quality of care that you provide in the dental department in general or in your wings particularly, how do you feel about it?

Interviewee: I can say that we try but it is not good; I cannot say that the quality is good (both smiling). We try but because of what I told you before related to the lack of some materials, and also when one of us who should also be here is not here, it disturbs the team work and increases the workload of the others.

Interviewer: I understand. It means that the quality of care is impaired by the lack of materials and staff.

Interviewee: Yes. Those are the challenges

Interviewer: When one of the equipment gets damaged like the dental chair, the sterilizer, and the compressor, those important machines like x-ray's; how is it? Does the administration hurry up to repair it?

Interviewee: Concerning the repair of equipment, this facility has a service of maintenance. When there is a problem, they come immediately and check what happened, unless when they cannot manage or if something must be bought through the administrative processes; but for

the maintenance, the service is rapid. When you call them, they immediately come and do the repair because otherwise, the work would stop.

Interviewer: Yes. It means that they don't delay, they care for it and value the service?

Interviewee: Yes, this is how it is.

Interviewer: You told us that the polishing paste is there and that it works properly, it doesn't happen that there is a stock out of it for a long time?

Interviewee: The challenge here, at one moment materials are available and at another moment they are not there. You can make a request today and wait a whole year for delivery. Meaning that one year from now, you will be working properly but you can spend a whole year or five months without working. We had a chance to start the present year with all the materials but currently they are finishing and the challenge has already started. We are doing treatments but in the months ahead, they will ask even the students to bring their own materials. These past months we didn't even ask the students to bring their own materials because we had plenty of them but now the stock is emptying slowly by slowly. This is the challenge; you can make a request today and wait a whole year for delivery. They keep telling us that materials are on the way; we clinicians cannot know the reasons behind that.

Interviewer: When you are doing treatments, do you feel secure especially about the risk of contracting an infectious disease?

Interviewee: I don't feel secure. You know that dentistry needs assistants but since the number of staff is not sufficient and the materials also are not enough, that is really straining on us. Think about treating a patient while holding the suction yourself, or isolating the area yourself. Sometimes the infection control fails and you ask the patient to hold the suction for you. You understand that it is not correct but when we try to tell our colleagues, they reply that they have been working like that for a long time and nothing happened. This is routine and routine should change as dentistry is developing. Maybe people were doing that because they were few. If we could have at least two dental assistants, the service would be even quicker. Think about treating a patient and cleaning the chair yourself before receiving another patient. If the assistant was there, they could help you and you would keep working without delay. The infection control is not fully respected because you are working in chaos. You can even be injured by endodontic files because you are rushing in order to fix an appointment and receive another patient. Even dental cleaning is not performed as it should because of that chaos.

Sometimes you clean only where it is obviously dirty but it is not correct. The protocol of infection control should be respected here because this is a teaching hospital. This environment should be similar to students' usual learning environment at school and it doesn't require a lot of things.

Interviewer: Apart from that aspect, are personal protective equipment available?

Interviewee: Yes, this facility has PPE. This is the only thing that is not missing. Not only for the clinician but even if you wished, you could put it on patients and they would be protected a hundred per cent. Apart from disposable gowns, face shields of really good quality are also there. However, sometimes you are lazy to wear it and prefer to use eye goggles, because it requires its pieces to be put together in many stages, but it really protects. For the gowns, our dental service has many of them even more than the theater. We don't have a problem related to PPE except for an individual who is lazy or negligent and fails to wear it.

Interviewer: Now, what could be done in order to ease your work in general?

Interviewee: First of all, increase the dental staff. Even if they could recruit at least two dental assistants, in the same way that they bring someone for the sterilization of instruments while we all are able to do it ourselves, these persons would assist clinicians because, even if in this wing there is a dental surgeon and a dental therapist, the dental therapist is not an assistant of the dental surgeon. Both practice independently, each one of them has duties. We do patients' consultations together and share the tasks, then rotate on the dental chair for the treatments but still that challenge of someone who could assist us remains. It would be better to have a third person. Secondly, all staff members should be monitored to check if the workload is equitably shared, instead of pushing all the work to the one who is very active. You can find that some people are not willing to be as active as the other, while this would smooth the teamwork and quicken the work. You can be thinking that your colleague is around while they have gone without even telling you. That is so frustrating.

Interviewer: I understand. Do you have enough dental chairs?

Interviewee: Dental chairs are not enough for here. In fact, only two are fully functioning. Clinicians working in other wings have to ask you for support. You have to exchange wings or they tell you that after finishing the treatment you were doing, they will bring their own patient and in between you will be waiting. Such a big hospital should increase the number of chairs

because if there were more dental chairs, the service would be improved. If we could get another dental chair in this wing, with a dental assistant, everything would be better.

Interviewer: A dental chair for every practitioner and a dental assistant?

Interviewee: Yes, even if the dental assistant is only one because some procedures are simple, they don't require an assistant. He/she would help us in the planning and in serving needed materials.

Interviewer: Now, if there was an application which would be installed in patients' telephones in order to give oral health education in general, what impact that would have on your daily work?

Interviewee: I think that this application would be an answer. It would be an answer to some of our problems like the fact of not educating patients. Oral health education is like the backbone of dentistry. If people were well educated about oral health, even treatments would stop. Oral health promotion is not such complicated. It requires only that the patient is informed, is updated, always having an access to the information they need. There is a patient for whom you may start a treatment like root canal, it is safe to do it during pregnancy and you tell her to come back. After leaving you, she meets another person who tells her that it is not allowed to treat a tooth during pregnancy. She then stays home and comes back after delivery when the infection has become severe and abscesses are visible. In that case, she will be obliged to take strong antibiotics which are not allowed for a breastfeeding woman. If that woman was updated and could access the information from that mobile application, it would help her. Patients should be made aware that it is important to install the dental mobile application in their telephones in the same way that they have to pay for medical insurance. That marketing and population mobilization is the responsibility of the owners of the application. On our side it can be helpful in that way that it would be providing information that we don't provide and change the patients' understanding linked to the fact that we don't have time to explain to them. The application would be important in solving some of our problems.

Interviewer: You kept coming back to time, time, time. Do you think that this application can reduce the time you used to spend with patients?

Interviewee: Yes, the application can reduce the time we spend with patients. As practitioners, we can give some oral health education to patients and then tell them to go and watch the information from the application, telling them to ask clarifications of what they wouldn't fully

understand. Since it would be helping us, we also would help it by referring patients to it. These are the two areas in which the application would be useful to us i.e. reducing the time we spend with patients and giving a deep oral health education, better than we do.

Interviewer: And those two areas are really important!

Interviewee: Yes, they are very sensitive.

Interviewer: You told me that whoever needs scaling and polishing you can do it for them. How many patients can you scale based on the number of instruments you have?

Interviewee: That is where the challenge of instruments and materials comes in. With much effort, we can do scaling for two patients per day. We don't send anyone back but we don't do it the same day they come except for whoever has a problem which cannot wait. In that case, we do extra working hours. We have two scaler tips used by all the dental practitioners, that is why the tactic we use in our wing is to ask the patient to come early in the morning at 7:00am when others are still preparing to start working. We can do scaling for two patients so that the tips can be sterilized for whoever else needs to use them. That means that if you want to treat a third person, you will have to wait that all those who were on appointment in other wings be treated. That is why we accept to go beyond normal working hours, waiting that the tips are sterilized in order to serve that patient.

Interviewer: That is really good, it shows that you value that treatment.

Interviewee: Yes, we do our best.

Interviewer: You told me that there are only two ultrasonic scaler tips, what about manual scalers. Are they enough?

Interviewee: Yes, manual scalers are available but many patients who come here at here has advanced periodontal cases on which it is not good to use manual scalers. Sometimes you do scaling in order to try to keep mobile teeth in place; teeth might be so mobile that if you use manual scalers on them, they can be exfoliated. But on the other side, I can be doing a root canal treatment for a patient and find that he/she has some calculus on one or two quadrants. In that case, instead of wasting time looking for the ultrasonic scaler, I bring a manual scaler and remove them in order to prevent infection of the treated tooth. That is how I use manual scalers but they are even too many, more than enough for me (smiling).

Interviewer: How many patients per day can be served using the manual scalers?

Interviewee: I think that even 50 patients can be served. They have a lot of scalers and curettes. The only problem would be insufficiency of staff. Some instruments are still new and packed in their pouches.

Interviewer: You told me that if more staff would be recruited among which dental assistants, and dental equipment also availed, the work would be easier; is there anything you want to add on that?

Interviewee: Well, if these things are there, the only thing needed would be a good work planning and the service would be quick. That is what private dental clinics have in advantage over public ones (smiling). Since they are doing business even though they are also helping patients, materials are covered in the price of the treatment but here it is different. Community based health insurance pays a little amount of money compared to the materials used for that procedure. That is the challenge of public dental clinics. Otherwise, if we were also well equipped, we can do better and patients would not need to go in private.

Interviewer: How many patients can you receive in this wing per day? Around twenty-five?

Interviewee: Patients we receive for consultations cannot go beyond thirty and those we treat are around twelve. When there are many cases for extractions because we consider that act as simple, we can go up to 15 treatments. However, since there are many root canal treatments which go together with filling the tooth and which takes a long time, we plan like ten patients but they are even so many.

Interviewer: I understand. Thank you so much. The information you have given us is very important.

Interviewee: Thank you too. I also found the questions very relevant.
