# Supplementary material for: Evaluation of oral health services and challenges faced by oral health practitioners working in Nyarugenge, Rwanda
Source: PLoS One. 2024 Aug 19;19(8):e0309127. doi: 10.1371/journal.pone.0309127 (PMC11332939; doi:10.1371/journal.pone.0309127)
Supplement: S1 Dataset — (ZIP) [file pone.0309127.s001.zip › dataset/Dataset qualitative interview transcript/PARTICIPANT (5).pdf]

INTERVIEW WITH PARTICIPANT 5

Interviewer: **Thank you for accepting that we have this interview. We are conducting a research about the challenges that dental practitioners might be facing when they are treating patients. This research is done here in Nyarugenge district. We would also know the role of an application which would be put into the phone, in educating patients about oral health. We thank you because you accept to interact with us about that and as we told you, research is confidential. Your name will not appear anywhere and no one will be able to say that this information is coming from this person. There is no wrong answer, every answer is important. We would like that you answer freely and give us all the information you have and we assure you that this will be useful in the present research when they will be compiled with what others will tell us. It will also be important for oral health in general. because every information will be kept confidential and We are requesting your permission to record your answers.**

Interviewee: No problem

Interviewer: Thank you so much. Now tell us, how do you feel about your work?

Interviewee: Thank you. In my work, challenges are there and they are not even few. Those challenges are especially linked to the understanding of people who come to us. Many are not knowledgeable about the oral health care. This needs that we put a lot of efforts in order to educate them. First of all, they don't know about oral conditions. They neglect them and don't consider them as serious diseases. Especially when the patient is not yet feeling pain, they don't think that they are sick, they don't give it the importance. Most of the time, they come at a later stage, when the disease has already gone beyond repair.

Interviewer: Based on how it is, are you pleased to do that job? Is your job tiresome, do you sometimes have to rash and work very quickly in order to clear the line? Are there some challenges? Feel free and tell us about how it is.

Interviewee: Yes I enjoy it, I enjoy it a lot because I was happy studying dentistry. In addition, this work is very important because treating oral conditions is one of the most important things that people should know because it helps people suffering from those diseases to be cared for, in an appropriate way not in the way people used to say that the only treatment of teeth is to extract them, not considering that a tooth is a body organ as all other organs and that removing it is not a problem. I enjoy my work because it helps me to explain to my clients how teeth are

body organs at the same level with the others and that they are very important for the body. Keeping teeth inside the mouth as long as possible and in a good state is very important for the population and for all of us. The only way to get that information is from health facilities because people are not informed about dental issues. When they come to us and that we explain to them, they can become aware that teeth are very important and that it is necessary to keep them and to care for them. I enjoy doing that even though we meet challenges like when we have many patients. We need enough staff, enough equipment, instruments and materials in order to offer a service of good quality to people who come to us.

Interviewer: You didn't tell us if your work is tiresome, if you really have many patients, how do you feel about it?

Interviewee: Well, the work is tiresome but when you come in the morning, you make a daily plan based on materials you have and the expected patients, saying that this is the number of patients you may receive and give them a service of quality. Yes, it is tiresome but we manage.

Interviewer: Do you sometimes need to rash in order to clear the line of patients?

Interviewee: It is not necessary to rash, even if I treat few but treat them well. That is what is better for me; I can give an appointment to others based on the number I received.

Interviewer: How many patients can you receive per day and treat them?

Interviewee: It depends on the cases of patients who came, on what I have to do for them. I never go beyond fifteen patients per day but all of them don't need a same treatment but different ones.

Interviewer: Does it mean that only fifteen patients come or some of them have to return home without treatment when they exceed that number?

Interviewee: When they are more than fifteen, I only receive fifteen because that is my daily goal; others are given an appointment.

Interviewer: If sometimes they are many and you have to send some of them back, were you expecting to receive such a great number of patients?

Interviewee: I was not expecting it but as medicine is advancing, many people are becoming aware that it is important to look for treatment; that is why on some days, they are many. It depends on people's understanding. When you treat patients, they become ambassadors and

tell others that there is someone who treat oral diseases and people may come in big numbers but one is not expecting that.

Interviewer: Sometimes when something happens to you which you were not expecting, it frustrates you. How did you react to that?

Interviewee: At first you are somehow disoriented but you try to stay calm in front of patients and take other strategies in order to help those who come to see you. You also explain to them and appease them.

Interviewer: Thank you so much. We want now that you tell us about **giving oral health education to all patients who come to you. Tell us, is it really possible? Is it not possible? How do you see it?**

Interviewee: When they come like at the same time, you go and do a mass oral health education. I explain to them the importance of coming early for dental treatment, about dental check-up even when someone is not suffering so that they may learn the status of their oral health, but even individually in the consultation room, I educate them based on their particular cases.

Interviewer: It means that it is possible for you to do oral health education to every patient, before you treat him/her.

Interviewee: Yes, I do.

Interviewer: What are the main topics you tell them about?

Interviewee: Thank you. First of all, I talk about how to do oral hygiene, then I talk about nutrition, dental check-up because many come at late stage when it is even too late to save the tooth. When they know that you can go for dental check-up even before feeling any pain, it is helpful for them.

Interviewer: Do you have some didactic materials or you only talk to them?

Interviewee: No, I don't know, maybe it is because of the structure which is still low, we don't have didactic materials.

Interviewer: No tooth brush and no model?

Interviewee: We don't have them.

Interviewer: No dental floss? Nothing?

Interviewee: Nothing.

Interviewer: What challenges do you meet when you want to do oral health education?

Interviewee: The first challenge I meet when you want to do oral health education is lack of didactic materials as we were talking about, it requires that I use only gestures so that I might explain to them like how to brush teeth. We cannot be sure that the person really understood,

Interviewer: You said that this is the first challenge. What is the second?

Interviewee: Another challenge is that most of the patients want to be treated quickly and go home. If for example one came for extraction, he/she tells you that what they need is extraction, do it for me and don't waste my time. During that time, even other patients on the line start complaining about delay. The waiting time factor also comes in as a challenge. Those two are the main challenges, didactic materials and time.

Interviewer: Now, tell us about scaling and polishing of teeth. How is it? Are there many patients who need it? Is it possible that you provide that treatment to every patient who need it? Tell us about it.

Interviewee: Thank you. Scaling and polishing of teeth is good and many, many patients need this. However, many don't even know that they have that problem, they come for a different problem. They don't even know that it is important that their teeth be cleaned professionally but the majority of patients who come to us need that treatment. I can say that 80 or 90% of them need scaling and polishing. Another thing is that many of them have some beliefs that it can spoil the teeth. They accept rumors and don't know the importance of it. Their understanding is still poor.

Interviewer: Let us suppose that they understand the importance of it and that all of them need that treatment, what challenges can you meet in that? Can you receive all of them and offer them that treatment?

Interviewee: I can meet the challenges of instruments which are not enough compared to the big number of persons who need it.

Interviewer: Tell us now about the sterilization of instruments.

Interviewee: There is no problem about the sterilization of instruments. I have a sterilizer.

Interviewer: I asked that question because you told me that instruments are few, is it not possible to sterilize them in between? How is it?

Interviewee: Yes, it is possible, but we don't think about it. In addition, you can treat the first patient, the second and even the third, finding that all of them need scaling and polishing. This becomes very challenging, especially that this was not their chief complaint, they were not even aware of it. However, we shall try and see if we can sterilize in between.

Interviewer: The instruments you have can be used on how many patients daily?

Interviewee: They can be used on five patients.

Interviewer: You told me that when you see like three patients who need scaling and polishing, it becomes challenging. How does it become challenging? Can you explain me more on that?

Interviewee: Sterilizing instruments?

Interviewer: You just told me that when you realize that the first, second and third patient need scaling it becomes very challenging. How? What do you want to say?

Interviewee: The reason why I said that is because if I have to do an extraction for the first patient but on examination I realize that he/she has also calculus requiring dental scaling, I prioritize maybe to start with scaling and do the extraction after; it is challenging if I do the same for the second and the third patient, and going for sterilization, because I still have other patients outside waiting for me who also need that I help them.

Interviewer: Did it ever happen to you to miss an instrument for scaling and polishing because it is not sterilized?

Interviewee: No, it never happened because before starting, I first check if I have sterile instruments.

Interviewer: You mean that you have never missed an instrument either for extraction, scaling or filling of teeth because they are not sterilized?

Interviewee: I cannot miss them because in the evening when I finish work, I clean them and sterilize them so that in the morning, all the instruments are ready.

Interviewer: Thank you so much. Now, when you complete the procedure, what happens? You have received a patient, you treated him/her, do you have time for instructions? Tell us about that.

Interviewee: When I am still with the patient?

Interviewer: Yes

Interviewee: When I am still with the patient after the treatment, I give post-treatment instructions based on what I did for them.

Interviewer: And is there any challenge in that which you can tell us about?

Interviewee: No challenge

Interviewer: Even about the time?

Interviewee: No, there is no challenge.

Interviewer: When you think about the quality of oral care that you provide here, how do you find it? Are you satisfied with it?

Interviewee: The quality of care we give is good, I am happy with it

Interviewer: When you say that the quality of care is good it means that nothing should be improved on what you do? Are you fully satisfied?

Interviewee: It is good because when materials are finished, they buy them for us. Everything is ok.

Interviewer: It means that you are able to offer all the services that your patients need.

Interviewee: Yes

Interviewer: Sure? It cannot happen that a patient might come and be referred elsewhere because you don't have required equipment or materials, even when you were able to do it.

Interviewee: That happens. There are some instances when you examine a patient and find that I don't have required items for the treatment. In that case, I send them to the hospital.

Interviewer: And on your side, you were able to perform that act; you refer only because of equipment or materials?

Interviewee: Most of the time, we are limited by our scope of work. Whatever is beyond our scope of work, I don't do them, I refer the case to the hospital.

Interviewer: No, I was asking about what is in your scope of practice.

Interviewee: What is in our scope of work, I do it no problem.

Interviewer: Tell us about dental equipment? I don't mean the instrument but the machines. How is it?

Interviewee: Here we don't have dental equipment. We have asked for them but we haven't yet received them. We only have few of them but not a fully equipped dental clinic.

Interviewer: Can you tell us the ones you have?

Interviewee: We have the ones for extraction and scaling but nothing for dental fillings.

Interviewer: It means that you only have consumables but no equipment like the dental chair, compressor, ultrasonic scaler?

Interviewee: We don't have any of them. We sent a request to the Ministry of Health and we are still waiting.

Interviewer: What happens when one of the instruments get damaged. Is the administration eager to repair or to replace it?

Interviewee: When that happens I inform the administration and they request someone form the district hospital. There are staff there who come and help us.

Interviewer: Do they respond to your request quickly or it takes time?

Interviewee: No, they provide quickly

Interviewer: It never happens that they can delay and make you to spend days without working?

Interviewee: No, they don't delay.

Interviewer: Very good. You told me that sometimes you do dental scaling for your patients; do you have polishing paste?

Interviewee: We don't do polishing.

Interviewer: You never do polishing?

Interviewee: Never.

Interviewer: Now, when you are treating…. (someone came and called the participant who went and spent more than one hour before coming back due to some visitors). **You had told us that you don't have the dental polishing paste. Now tell us about how secure you are while providing the treatment.**

Interviewee: Thank you. When I am treating, I feel secure but it would be much better if I had a working partner. That would make someone to feel more secure.

Interviewer: I was referring especially to the fear of being contaminated during treatment.

Interviewee: No, I am not afraid of that

Interviewer: Why?

Interviewee: Because I protect myself, wearing gloves, face masks, I am not afraid.

Interviewer: What do you use to protect yourself?

Interviewee: I use face mask, goggles, medical coat, and gloves

Interviewer: Do you have any head cover?

Interviewee: Yes, I also put a cap on my head

Interviewer: It means that in that area, you feel fully protected. Any challenge on that side?

Interviewee: No challenge

Interviewer: Is that personal protective equipment always available? You can never miss them?

Interviewee: No, we never miss them

Interviewer: When they finish, what happens?

Interviewee: What can miss is only the head cover but others like the medical coat is always there. If you miss something, you help yourself with what you have and you must be very careful during treatment.

Interviewer: How long does it take when you tell them that you need a certain material before getting it?

Interviewee: It can take like three days or one week. I make sure to tell them before everything is finished, I still have what to use for emergency cases.

Interviewer: What do you think might be done in order to make your job easier?

Interviewee: What can be done to ease my job is to avail enough dental materials. Secondly, one staff is not enough, it would be much better if they were two.

Interviewer: What do you mean by availing enough materials. Which ones are not there?

Interviewee: Consumables are most often missing like dental cartridges. They can even be missing in the pharmaceutical stores. Apart from consumables, we don't experiment any other shortage of items.

Interviewer: You are only telling us about consumables while above you told me that you don't have any dental equipment; don't you think that if you had equipment it might ease your work?

Interviewee: Of course, if it is possible, there are much needed. But since they are not there, we try our best to use consumables on a daily basis, reason why if they are not available, it is a big challenge.

Interviewer: What is this helping you? (pointing at an old equipment present in the office)

Interviewee: This used to help me in dental fillings but it is only one and that is not enough

Interviewer: Is it well functioning?

Interviewee: Yes. But it is not enough at all (smiling)

Interviewer: What do you use when you want to do scaling?

Interviewee: I use manual scalers.

Interviewer: Now, if there was an application which would be installed in patients' telephones in order to give oral health education in general and how to perform oral hygiene, what importance that would have for you?

Interviewee: If that application was available, it would help me to gain time.

Interviewer: How. Explain more how it would help you to redeem time

Interviewee: The time I used to take in order to explain to the patients, since that application would be explaining to them, I would be doing the other work. It would help people and it can reach many people at the same time. In addition, all people get the same important information. Since it is possible to replay, it would help someone to understand better.

Interviewer: And what importance that application might have in terms of dental health and oral health in general?

Interviewee: Not only here at the health center where it can be used for mass education, but since it will be installed in personal telephones where everyone can access it wherever he/she is, they can watch at their convenience, when relaxed, without stress and that would help them to understand better. That would raise the awareness of people to care for their oral health and to look for dental care earlier, without waiting to go for healthcare when the tooth can no longer be restored. The application would allow them to understand that prevention is better than cure. They would know how to brush their teeth and would help her to prevent oral diseases.

Interviewer: Thank you so much. Now, which advices can you give so that all the materials and equipment needed in teeth scaling and polishing are available and useful for you?

Interviewee: To be available, the administration of the health center should make a request to the concerned people who must provide them.

Interviewer: You told us that you use manual scalers, are you comfortable with that?

Interviewee: No I am not comfortable because it takes time and is very difficult.

Interviewer: And which advice can you give in that matter of scaling and polishing so that they become easy for you?

Interviewee: I would be happy if they bought for me new and modern equipment

Interviewer: Like which ones?

Interviewee: Like ultrasonic scaler

Interviewer: I saw that you were not willing to pronounce its name as if it was your father-in-law (all laughing). What advice can you give now so that your work in general could become easier?

Interviewee: First of all, if that application could be availed, it would be good. Secondly (hesitating), if all the needed equipment and consumables for a good dental clinic could be available, it would help me so much.

Interviewer: Nothing else?

Interviewee: We cannot forget to mention that more dental staff is needed so that the number of patients we serve might increase. This would help them to get dental services nearby instead of going far, at the hospitals.

Interviewer: Do the few dental instruments that you have, like those manual scalers, function properly?

Interviewee: Yes, the ones I use are effective, no problem.

Interviewer: What do they reply to you when you tell them to buy equipment?

Interviewee: We are waiting. They made the request and sent it to the Ministry of Health, we are waiting

Interviewer: You have not yet got the feedback

Interviewee: Not yet

Interviewer: Thank you very much. All the information you have given to us is good and valuable. If my colleague also wants to ask you something

Note taker: No more questions. You answered well. Thank you

Interviewer: Thank you so much and wish you success in your work.

Interviewee: Thank you too. I appreciate.
