# Supplementary material for: Evaluation of oral health services and challenges faced by oral health practitioners working in Nyarugenge, Rwanda
Source: PLoS One. 2024 Aug 19;19(8):e0309127. doi: 10.1371/journal.pone.0309127 (PMC11332939; doi:10.1371/journal.pone.0309127)
Supplement: S1 Dataset — (ZIP) [file pone.0309127.s001.zip › dataset/Dataset qualitative interview transcript/PARTICIPANT (6).pdf]

INTERVIEW WITH PARTICIPANT 6

Interviewer: Thank you for accepting that we have this interview. We are conducting a research about the challenges that dental practitioners are meeting in their practices and the importance of an application which would be put into the phone, in educating patients about oral health. We would like that you answer freely because every information will be kept confidential and there is no wrong answer, every answer is important. We are requesting your permission to record your answers so that we don't lose anything of what you will tell us.

Interviewee: No problem

Interviewer: Thank you so much. Now, the first question goes like this. How do you perceive your work currently? Are you happy with it? Is it really tiresome? Do you sometimes have to rash and work very quickly in order to clear the line? Are there any challenges? Feel free and tell us how it is.

Interviewee: Challenges are there especially when you compare the number of dental practitioners and the number of patients we receive. Usually the target of establishing referral hospitals was to treat cases which cannot be treated in other health facilities and according to standards; to reduce the number of patients but offer them quality care. We cannot attain that objective because in district hospitals and in health centers they don't have the ability; they refer almost all the patients to us and when you compare with the number of clinicians we have, it is very challenging to manage that. Practitioners are overloaded.

Interviewer: What impact does that have on your feelings?

Interviewee: First of all, I become stressed because I see patients who need services. Telling someone to come back after one month is very frustrating. You would like to serve them immediately but due to the capacity you have, you are obliged to give a rendezvous one month later. You understand that we cannot solve patients' problems a hundred per cent.

Interviewer: Were you expecting to receive such a great number of patients on a daily basis?

Interviewee: I didn't think that they can be such much to this level, but I try to adapt to the situation even though it should not be like this, considering that the target for referral hospitals was totally different. Since we are in a country under development, we adapt to the situation

even though it is hard. Sometimes you spend the night thinking about that, you carry your work at home. Even sometimes during the weekend, you carry the registers at home in order to do some statistics from home. You understand that it is tiresome, you don't have time to rest.

Interviewer: Now, tell us about giving oral health education to all patients who come to you. Tell us, is it really possible?

Interviewee: Usually that is one of our primordial tasks which should be performed because we even submit a plan on how we shall do mass education. However, sometimes when you see the number of patients, you realize that it is somehow impossible and we prefer doing a chairside oral health education which doesn't offer enough information. Sometimes you tell them something and when they get outside, they hear rumors and prefer to erase what you had told them. But if the education had been done in common, they would have asked questions and go home satisfied; it would be much better especially that this is one of our objectives.

Interviewer: It means that the challenge you have concerning oral health education is linked to the number of patients?

Interviewee: Yes, people are overloaded

Interviewer: When you manage to give oral health education sometimes, which are the main topics do you tell them about?

Interviewee: When you consider how all the people including even health professionals are still ignorant about tooth brushing, or they neglect to do it, telling you that they rarely brush at nights, you realize that they don't know the rationale behind tooth brushing. When you ask them at what time they brush, they tell you that they do it early morning, when they get up. Why do you do it at that time? Do you know why you need to brush your teeth? We ask them and they reply that they brush in order to remove the bad odor from the mouth. You understand that they don't know why they brush. After brushing, they immediately drink porridge, at noon they take lunch and at night they eat and sleep without brushing. They really don't know why they have to brush their teeth. That is why we insist on that topic because the bigger number of patients we receive are suffering from dental caries, meaning that brushing habit is still at a low level.

Interviewer: When you manage to teach them, do you have didactic materials or you do it only in theory?

Interviewee: I don't have didactic materials but I have images. Sometimes students bring pictures with them or sometimes they even have didactic materials. That is when I have the chance to use didactic materials, when I have many students in clinical placement.

Interviewer: Yes. Now, when there are people who need scaling and polishing of teeth, do you think it is possible that you provide periodontal treatment to every patient who needs it the same day they came?

Interviewee: It is not easy for us, but at least you inform them and make them aware that that treatment exists. You cannot do it for them immediately, you give them an appointment. However, that education is not provided in common, it is between the patient and the practitioner. I cannot know exactly what someone in another wing has taught to the patient. I don't have full information on that (smiling).

Interviewer: Are there many patients who need that treatment?

Interviewee: So many. They even come looking for that but it is challenging for us due to the number of patients.

Interviewer: Tell us now about the sterilization of instruments, how is it?

Interviewee: Concerning the sterilization of instruments, we don't have a big problem because we have the main hospital sterilization unit and here in the dental service, we initially had three sterilizers but one of them got spoiled. This was a donation and it was big and able to sterilize at any time, but currently we have two small, so that any instrument you might need would be ready and available to be used after one hour or after a given time depending on the sterilization time of items.

Interviewer: It means that you cannot fail to treat a patient due to lack of a sterile instrument?

Interviewee: No, but we can delay like for one or two hours due to the number of patients and the instruments we need. It is true that instruments are not sufficient but the sterilization itself is not a problem.

Interviewer: Sure? How many patients can be treated by scaling and polishing per day, based on the instruments you have?

Interviewee: Two patients.

Interviewer: Two patients. Why? Is it because of time or of available instruments?

Interviewee: Because we currently have two working scaler tips, others have been damaged. It means that when we treat two patients, others can get a chance to be cleaned after a period of two hours of sterilization. However, this situation is not very common because of the big number of patients we have.

Interviewer: What about manual scalers? Do you have them?

Interviewee: Manual scalers are there, they are even many, but we usually use ultrasonic scaler.

Interviewer: When you finish to treat a patient, do you have time to give post-treatment instructions?

Interviewee: We don't take enough time; we tell them in rush. This is not how we should do that and this is linked to the big number of patients. It is only when we see that the patient is at risk that we take time to educate him/her.

Interviewer: What about post-treatment instructions related to what you did for them?

Interviewee: The instructions are mandatory; the patient cannot go without instructions. If we did scaling for them, we tell them when they should come back for the next session. Yes, for the instructions, we give them.

Interviewer: Yes. Tell us briefly about the quality of care that is provided here? Are you happy with it? Is it really good?

Interviewee: The quality of care? I cannot say that it is a hundred per cent good but it is like at seventy per cent. We perform at least 70% of what we should offer to the patient.

Interviewer: Could you explain to us on which you base that percentage?

Interviewee: I base that percentage on the procedures we do. The ones we are not able to provide are not so many compared to the number of patients referred here. If for example a patient gets an extraction of teeth and goes back without dental prostheses while this is a referral hospital, you understand that this treatment is not complete. If they got accidents or tumors, they will wait long before the treatment because until now we have only one specialist dealing with these patients. Even though that patient ultimately will be treated, this can take

like from six months and above; even to one year. That is why I give that average. Yes, the treatment will be provided but very late.

Interviewer: When one of the equipment like the dental chair, the compressor, or the sterilizer gets spoiled or is not functioning well, does the administration hurry up to repair it? How is it?

Interviewee: We have a service of maintenance which really tries. Every service has an appointed technician that we call in case there is a problem. When that one is not on duty or is on annual leave, he/she gives us another telephone number of the person to inform. We don't have a big gap on that side.

Interviewer: Sure? What happened to that sterilizer? Why were not they able to repair it?

Interviewee: The sterilizer was new and one of the inside pieces burnt. It cannot be repaired without buying a spare part, and it must be brought form overseas.

Interviewer: Was it still in the guarantee period?

Interviewee: It was a donation

Interviewer: But in general, do you think that the dental equipment is valued? When you say that one of them doesn't work properly do they repair it quickly?

Interviewee: Yes, they do. We have a letter of appointment of the technician allocated here. In general, service of maintenance is working well.

Interviewer: Let us now talk about the polishing paste. Is it also valued so that when it is not available they buy it quickly?

Interviewee: Polishing paste?

Interviewer: Yes, when it gets finished.

Interviewee: Currently the tender process is smoother. They allowed us to buy from four more pharmaceutical stores among which BUFMAR in case we need something urgently, in order to avoid stock outs.

Interviewer: It means that you have a polishing paste which is working properly?

Interviewee: Yes, we have

Interviewer: At some places they told us that they don't have it or that the one they have is of poor quality. For you is it of good quality?

Interviewee: We don't have any complaint about it

Interviewer: How secure do you feel when you are treating patients, especially on the side of the risk of contracting an infectious disease?

Interviewee: We gained advantage from corona pandemic because personal protective equipment has been availed. We have gowns, face masks, and face shields. There is no problem on that side except for practitioners' understanding. Some of them are negligent and don't protect themselves but materials are available.

Interviewer: It means that whoever wants to wear PPE would get everything? Even head caps?

Interviewee: Yes

Interviewer: As a referral hospital, do you have the cover sleeves for protecting the light or hand pieces? Do they sometimes avail them?

Interviewee: I have never seen them

Interviewer: Have you ever tried to request them?

Interviewee: Yes, we requested them but until now we have not yet received them.

Interviewer: Which advices can you give in order to make your job easier?

Interviewee: If I would never experience a stock out of consumables and also if the number of patients would be adjusted. Sometimes you don't know how to give appointments due to the number of patients in front of you. If I could receive an appropriate number of patients that I am able to manage, this would make my work much easier. I would do my treatments better and have the time for individual oral health education. I would not provide treatment in rush in order to clear the line. Most of the time we are offering more quantity than quality in care.

Interviewer: Which factors do you think might reduce that big number of patients?

Interviewee: I don't know if it is due to the tender process or the overall poverty we have in the country. They should equip district hospitals with enough materials and equipment, so that only special cases would be referred here.

Interviewer: Concerning staff, you don't have any problem? Are they enough?

Interviewee: So far so good. There is a problem which is common in public clinics; in private clinics they have assistants and in dentistry there should be at least four-hands. Here you are the only one either to receive the patients, to treat them or to assist yourself in needed materials. That can lead to poor infection prevention and control (IPC). I always wonder why they don't provide dental assistants in public health facilities.

Interviewer: Have you ever tried to ask for them?

Inter*viewee: I think that even at the school of dentistry level or everywhere in Rwanda, dental assistants are not trained. That training should be provided because in other countries it is done.*

Interviewer: I know that at one time the plan was there but until now it is not yet implemented; we don't know when it will be.

Interviewee: You understand that this is a big challenge. If we have to work as four-hands and you are there alone, helping yourself, it is not correct. I don't know if it is because we are unable to express our needs. However recently I was busy fighting about x-rays because even though clinicians take x-rays, there are people who are in a better position to do that. We should be having a radiographer because for us we were shown only how to position the tube and how to process the film but we don't know the direction of rays. This is really a big challenge. After a lot of negotiations, they agreed to recruit one. That will also ease our work because we were taking x-rays ourselves except when we were lucky to have students but a student is a student, he/she is not one of the staff.

Interviewer: While you have not yet received trained dental assistants, have you ever given the idea to the hospital administration so that they train nurses for assisting you? When they train them, they do it well.

Interviewee: Before the recruitment of dental therapists, nurses were there. When we arrived, they took them away, saying that we should manage all by ourselves.

Interviewer: Keep trying to ask for them, maybe they will understand.

Interviewee: They say that nurses have not been trained to assist in dental and even that their number is not sufficient. The administration thinks that bringing a nurse to assist in dental is

like waste of human resources. Curious enough, the number of nurses exceeds the one planned by the Ministry of Health, but they are still struggling.

Interviewer: The reality on the field is only known by the team in place (smiling). Now, if there was an application which would be installed in patients' smartphones in order to give oral health education in general, what impact that would have on your daily work?

Interviewee: I think that would help because there are people who has smartphones but who are not informed about oral health. If you treat a patient and send them these education materials, this might change their minds. It would be easier to us to forward these videos in case we didn't have time to do it face to face. I don't know who will make sure that this application reach everyone through us but its impact would be positive. This would answer the question we had about providing oral health education to patients.

Interviewer: Do you think that this application can reduce the time you used to spend with patients teaching them?

Interviewee: I think that the application would be helpful in general. The time we took for oral health education would be shortened; the lack of didactic materials like a TV screen showing how to brush teeth or when people should go for a dental check-up, every six months, would be solved. All that would no longer be needed because they could watch from home on their telephones. It will also be good for us because we would be able to provide oral health education without having to take time and prepare teaching materials, we would have ready-to-be-delivered teaching materials. The application has a positive impact on both sides either for the patients or for the dental practitioner.

Interviewer: Which advices can you give so that all the materials and equipment needed in teeth scaling and polishing are available and adequate so that you might provide a better service?

Interviewee: On our side, we make an annual requisition, a list of everything we need. They try to deliver what departments need but not according to the time you needed them; they prioritize. Sometimes, they can bring items towards the end of the year like in October and in December then they tell you to make another list, while you have not yet received the items from the previous list. That is another challenge.

Interviewer: It means that for you, if they could avail materials, it would be much better.

Interviewee: Yes, it would be much better. I would be working comfortably instead of waiting the scaler tip to be sterilized for example, because it strains our nerves. When I lack instruments for treatment, this is so discouraging and the patient gets the service very late, causing the sterilization personnel to work extra hours.

Interviewer: But I wonder, why are there only two scaler tips? Have the others been used until they get damaged?

Interviewee: Yes, they got damaged. They are also on the waiting list of what we have requested. I had requested thirty but I have only two; but even those two are no longer functioning perfectly because they are overused.

Interviewer: Apart from that domain of prevention, of scaling and polishing, what would ease your work in general? Which advices could you give on that?

Interviewee: The clinicians need motivation, even a small one. If they could think about offering them a drink so that they don't fall into hypoglycemia, it would be good.

Interviewer: Thank you so much.

Interviewee: Thank you too.
