# Supplementary material for: Evaluation of oral health services and challenges faced by oral health practitioners working in Nyarugenge, Rwanda
Source: PLoS One. 2024 Aug 19;19(8):e0309127. doi: 10.1371/journal.pone.0309127 (PMC11332939; doi:10.1371/journal.pone.0309127)
Supplement: S1 Dataset — (ZIP) [file pone.0309127.s001.zip › dataset/Dataset qualitative interview transcript/PARTICIPANT (7).pdf]

INTERVIEW WITH PARTICIPANT 7

Interviewer: Thank you for accepting that we have this interview. We are conducting a research related to the challenges you might be facing in your daily work and about the importance of an application which would be installed in telephones and which would be delivering oral health education. There is no wrong answer, all the answers are important because they will be useful to us in this research and the research is performed in total confidentiality. Feel free and give us all the information. We would like to ask for permission to record the voices. Is there any problem?

Interviewee: No problem

Interviewer: Thank you very much. Now tell us, how do you feel about your work currently?

Interviewee: In general, it is ok concerning my work because I am able to examine patients and when there is something I cannot do from here, I refer them where there may receive treatment.

Interviewer: Based on how it is, are you pleased to do that job? Is your job tiresome? Do you sometimes have to rash and work very quickly in order to clear the line? Tell us about how it is.

Interviewee: I like my job and enjoy doing it. I studied this profession purposely and I like patients. This is why I don't have any problem whether patients are few or many, I treat them.

Interviewer: Yes, but when they are many and that you are rushing, what really happens?

Interviewee: When they are many, we sometimes don't do a deep intraoral examination and concentrate only on the chief complaint instead of doing a comprehensive oral exam. Even though maybe the chief complaint was a decayed tooth, you don't check if there are also teeth deposits or a tooth that needs extraction, or any other decayed teeth but which might still be restored. You don't check occlusion of any eventual device? When patients are many, you don't even have time to educate the patient.

Interviewer: Were you expecting to receive such a great number of patients on a daily basis?

Interviewee: Yes, because when you are working in the vicinities of the city of Kigali, patients are many, especially that they don't need a referral in order to come to the health centers. For that reason, you are expecting to receive a great number of patients. However, since almost all the health centers have a dental therapist, the number of patients is gradually reducing.

Interviewer: How many patients can you receive per day?

Interviewee: Apart from these current times where community based health insurance (CBHI) is not covering dental patients, I used to receive between 15 and 20 patients per day and when they were few, they could not go below 10.

Interviewer: And how many do you receive currently due to that fact that CBHI is not covering them?

Interviewee: I receive between 8 and 10 patients per month

Interviewer: Per month?

Interviewee: Yes, per month

Interviewer: Can you explain to us why the CBHI is no longer covering them?

Interviewee: The reason why CBHI is no longer covering them is because we used to have a dental chair which is no longer there. For that reason, CBHI is no longer accepting to reimburse dental treatments until the dental chair is availed again.

Interviewer: How many patients per month were you receiving when the dental chair was there?

Interviewee: I used to receive between 120 and 200 patients per month.

Interviewer: Let us hope that you will get it soon so that you continue helping patients. You told us that when patients are many, you sometimes don't educate them as it should. Expand a little more and tell us about educating patients concerning oral health, and oral hygiene in general.

Interviewee: Some people ignore that it is good to do oral hygiene or that it should be a daily lifestyle; they also think that when they don't have dental pain, other deposits should not be worried about. That is why I insist more on oral hygiene, telling them that if they could come early, these deposits might be removed and teeth would not need to be extracted due to these deposits. Another aspect I tell them is that when the tooth starts to become painful due to hot

or cold stuffs, they should go immediately for filling instead of waiting to go at later stage when the tooth will have to be removed. Again, many patients don't like dental fillings but it is because most of them are done on an advanced stage of dental caries, with the risk of developing secondary caries. That is why they say that dental filling is a waste of time. Brief, in my oral health education I mostly insist on oral hygiene and on seeking early for dental filling. I also teach mothers about canine mutilations which are very popular in these sub rural areas. I tell parents that it is possible that a child can develop some signs like diarrhea when they are teething but it is better to bring them here so that we help them instead of taking them for canine mutilations. Those are my main topics for oral health education.

Interviewer: It means that for every patient who comes to you, you find time to educate him/her about these topics?

Interviewee: It cannot be possible to educate every patient individually on these topics except now that I have few patients but usually, I examine all of them and after that, before starting treatment, I do mass education. It is very challenging to do oral health education considering that you are the only one who will treat all the patients.

Interviewer: What are the challenges linked to that?

Interviewee: The challenges are linked to working alone. When you are the only dental staff, you cannot teach the patients and treat them as it should; even telling them about other problems you discover of which they were not aware of. You are only oriented to their chief complaint.

Interviewer: You told us that sometimes you give them oral health education; do you have didactic materials or how do you do it?

Interviewee: I don't have didactic materials. Often I help myself with a tongue depressor and show them on my hand how to orient the tooth brush while brushing and the appropriate technique. We don't have models to use. When it is one patient during treatment, I tell them that they should brush teeth well, in order for these deposits to not accumulate again. I can even use a facial mirror to educate them.

Interviewer: Do you also mention about the dental floss when you are teaching patients?

Interviewee: Yes, there are some people who have teeth which are very close one another so that even when doing dental scaling you cannot penetrate in the interdental spaces. I tell them

how to prevent teeth deposits in those spaces by using a dental floss. Yes, I often talk about dental floss also.

Interviewer: Do you have the dental floss in order to show it to them?

Interviewee: No, I used to have it and to demonstrate for them but when it finished, I no longer show them but I tell them that it exists and show them the movements to perform when using it.

Interviewer: You just told us the challenges you meet in doing oral health education which are time and materials. Anything else?

Interviewee: No, challenges related to time, materials and working alone.

Interviewer: **Yes. Now, tell us about patients who come for scaling of teeth. You know that after scaling there is also need for polishing of scaled dental surfaces. Is it possible that you provide that treatment to every patient who need it? If it is not possible, why? Are there many patients who need it? How is it? Tell us about it.**

Interviewee: Many patients who come for scaling and polishing come with high expectations, thinking that you are about to whiten their teeth. Sometimes they have intrinsic stains which you cannot remove. In that case you need to tell them that only extrinsic stains will be removed but others will not be changed. Otherwise when you don't tell them, they expect teeth to be whiter after dental cleaning. However, only few patients come for dental cleaning. I could do an intraoral examination and tell the patient that he/she needs dental cleaning. They used to ask if there is no complication about that treatment due to some rumors they heard about it, I explained to them that the sensitivity they feel after scaling is often linked to gingival recession but that there is a toothpaste they can use for a certain period of time in order to relieve the sensitivity, instead of keeping these teeth deposits.

Interviewer: It means that whoever came to you, looking for dental scaling, you were able to do it for them without problem?

Interviewee: Yes, I did. Even at that time I had the polishing paste, I scaled and polished their teeth, and educated them about oral hygiene, especially that they were not so many. However, it was not possible to do it the same day of the consultation, I gave them an appointment because I had many patients. You cannot do extractions and scaling the same day when you are alone. I used to give an appointment for patients who needed scaling and feelings on the days where patients were fewer.

Interviewer: It means that you even did dental fillings?

Interviewee: Yes.

Interviewer: Tell us now about the sterilization of the instruments. How do you do it here?

Interviewee: After using instruments I soak them into a bucket with chlorhexidine solution. They then clean them and dry them. We sterilize them in the dry heat sterilizer at 180 degrees for 30 minutes. We used also to have an autoclave but they also took it.

Interviewer: Have you ever checked for the effectiveness of the sterilization?

Interviewee: We have not checked, especially that I have never had a patient with dry socket or any other post-operative infection. Whenever I sterilize instruments, I also renew instruments remaining in the container. All of them are soaked again and sterilized.

Interviewer: It cannot happen that you send a patient back home because there are no sterilized instruments?

Interviewee: No, it never happens. I have enough instruments.

Interviewer: You have instruments that can be used on how many patients who need scaling, or tooth extraction or dental filling?

Interviewee: I have instruments for like fifty extractions, ten dental fillings and four scalings before sterilizing again.

Interviewer: It means that you sterilize only once per day?

Interviewee: Yes, once per day.

Interviewer: You have never failed to treat a patient due to lack of sterilized instrument?

Interviewee: It is impossible

Interviewer: When you finish to treat a patient, what happens? Are you able to give post-operative instructions?

Interviewee: After an extraction, it is necessary to give instructions. Telling them that you put a gauze which they have to remove after 30-45 minutes depending on how much bleeding you observed. I also tell them like this: <don't insert the tongue or your finger because you would be infecting the socket, don't spit but swallow the saliva so that you don't continue to bleed, >. We explain all that to the patient

Interviewer: Is there any challenge about that, do you get time for it?

Interviewee: Giving post-treatment instructions is a must, whether you have few or many patients. When patients are many and that the line is so long, it can be challenging to give individual instructions; when that happens, I give them instructions in common before starting the treatments.

Interviewer: Tell us about the quality of care that you provide here? How is it? Are you happy with it?

Interviewee: Currently I am not happy with it because many patients come and return home without getting what they needed because of the CBHI; even when they have other insurance schemes, they don't get all needed care because there is no dental chair. People start to understand the importance of looking for dental treatment. Some even come asking specifically for dental fillings.

Interviewer: When someone comes looking for a certain dental treatment which you cannot provide, how do you feel deep in your heart?

Interviewee: I really feel bad because the service they needed and which I was able to perform cannot be offered because of lack of equipment.

Interviewer: You told us that you had a dental chair which has been taken from you; by the time you still had dental equipment, when one of them got damaged, how long did it take before they repaired it?

Interviewee: None of them got spoiled because they were new. We had them for less than one year. The suppliers still came after every three months to check how they functioned since the guaranty period was not yet finished.

Interviewer: Yes. And for how long have you been without any dental equipment currently?

Interviewee: Since COVID started, in 2020.

Interviewer: For two years now. What do they tell you when you ask?

Interviewee: When I ask to the administration of the health center, they tell me that the request has been submitted at the District office, that they are waiting for the feedback. Only the x-ray machine was left and it cannot be used alone.

Interviewer: But it can be useful!

Interviewee: The problem is that they left it but took the sensors. They even unfixed it from the wall. It cannot work.

Interviewer: The truth is that you don't have it either.

Interviewee: Yes, we cannot ask the health center authorities to fix the x-ray machine, they don't understand the importance of it.

Interviewer: What about consumables like the polishing paste, what happens when one of them get finished?

Interviewee: I made a requisition and they bought them

Interviewer: Without delay?

Interviewee: Yes, they are valued but I also made sure to make the request on time. As an example, where I hear that elsewhere they sometimes use local anesthesia from bottles, it never happened to me, dental cartridges were always available.

Interviewer: It means that here, dental services are very valued

Interviewee: Yes, they are. The only challenge we have is that our dental chair was taken. I was never denied me anything I needed.

Interviewer: Tell us now about your protection during dental treatments. How secure do you feel in terms of contamination by an infectious disease?

Interviewee: Protection is low especially when you consider this period of COVID. When you are doing a tooth extraction, even if you are wearing a face mask, you are not a hundred per cent protected. However, when you remember that this is what you have chosen to do, you hope for divine protection. I always wear a medical coat and face mask during treatment.

Interviewer: Apart from the face mask and the medical coat, which other personal protective equipment (PPE) do you have?

Interviewee: The PPE I have is made of face mask, gloves (I always wear surgical gloves), and the medical coat. Nothing more.

Interviewer: It means that your eyes and head are not protected.

Interviewee: I don't have any protection for the head but my eyes are protected because the face shield is there, I wear it together with the face mask and gloves.

Interviewer: Tell us now what can make your work much easier

Interviewee: Currently what I wish is to get dental equipment and be able to treat patients again. I don't need my work to be made easier because for now, I don't have enough workload given that there is no dental equipment. If they could bring back the dental chair which would allow me to serve the community, I would be happier and in case the patients become many, I would be happy if another dental staff was recruited so that we help each other.

Interviewer: Coming back to the application we were telling you about, which would be installed in peoples' telephones in order to give oral health education, would it have any positive impact on your work?

Interviewee: It is very useful because nowadays, people who don't have smartphones are few. Even if you are old and confined at home, one of your children might have it and would give you information about tooth brushing, professional cleaning in case there are tooth deposits, and early treatment for decayed tooth. People are currently interested in technology and receiving information from a phone can encourage them to seek for treatment at an early stage instead of coming late when the only possible treatment is to remove the tooth.

Interviewer: You told us earlier about the challenge of time, do you think that this application could shorten the time you used to spend with the patient?

Interviewee: Yes, the time would be reduced because the patient would be having some information concerning oral health, at arrival. They would be asking for explanations of some areas where they have not fully understood. You would clarify the information they would have instead of telling them about what they were not aware of. In that case, the time spent giving oral health education, struggling to tell them how is a tooth without didactic materials, that application would help more.

Interviewer: Sure? It means that for you, that application is very useful?

Interviewee: It is very useful. The person who thought about it should be honored (both laughing). I was even surprised to hear about that application. When you told me about it, I just realized that the information would be channeled to many people, better than through

television because TV is watched by few people. It would be easy to get the information through that application.

Interviewer: Yes. Which advices can you give so that all the materials and equipment needed in teeth scaling and polishing are useful for you?

Interviewee: What I have observed is that the polishing paste they bring currently is not effective. It doesn't remove stains on the teeth. After using it, you yourself observe that there is something remaining on teeth surfaces. Even though you don't tell the patient and they themselves see that you tried your best, polishing paste are of poor quality; either the ones I had here or those I find in other dental clinics where I go for part time job. When teeth have become stained by black tea, only a small layer of stains is removed. If the quality of polishing paste could be improved, it would be much better and patients would be more satisfied from the polishing you did for them and on our side, we would be more proud of the work we did.

Interviewer: Considering not only the polishing paste but also the instruments for scaling and polishing, which advices can you give?

Interviewee: It would be better if the tips for ultrasonic scaler were many because sometimes you scale few patients due to lack of instruments. There are other tips that are so big that they cannot allow you to remove deposits without traumatizing the gum. It would be better if they made them as thin as the tips for manual scalers. Only two out of the four tips can be used efficiently.

Interviewer: Which advices can you give in order to make your job easier in general? Forget the fact that currently you have been obliged to work less against your will. If the dental chair was there, if you had all needed instruments and materials, which advices can you give in order to make your job easier in general?

Interviewee: First of all, if the dental chair and all needed instruments, equipment and materials were available, so that when the patient comes and need any treatment he/she might get it, this would satisfy me. My wish is not to receive few patients but we should receive many patients and when need be, they would recruit another dental staff so that we help the population. Another thing is that they should stop the paperwork to record patients' information in the registers so that we do only electronic medical recording. It is time consuming to fill the registers, the patient's file and electronically. This is like triple work. The patient should be recorded electronically; this information would be noticed by the cashier

who would prepare the invoice for them. Then the patient would pay and come back for the treatment. In that case, the work would be easier, and the quality of care would be improved.

Interviewer: Thank you so much. Any question from the note taker?

Note taker: I think this is enough

Interviewer: We really thank you, all the answers you gave us are very important and they will be useful in this research.

Interviewee: Yes. All the best

Interviewer: Thank you
