# Supplementary material for: Evaluation of oral health services and challenges faced by oral health practitioners working in Nyarugenge, Rwanda
Source: PLoS One. 2024 Aug 19;19(8):e0309127. doi: 10.1371/journal.pone.0309127 (PMC11332939; doi:10.1371/journal.pone.0309127)
Supplement: S1 Dataset — (ZIP) [file pone.0309127.s001.zip › dataset/Dataset qualitative interview transcript/PARTICIPANT (8).pdf]

INTERVIEW WITH PARTICIPANT 8

Interviewer: Thank you for accepting that we have this interview. We shall try not to take a lot of time. As we told you, we are doing a research related to the PhD of our colleague. He would like to know the challenges that dental practitioners are meeting in their practices and the role of an application which would be installed in people's telephones in order to give oral health education. The impact that this application would have on your work. We would like that you answer freely because every information will be kept confidential and there is no wrong answer, every answer is important. We are requesting your permission to record your answers so that we don't lose anything of what you will tell us.

Interviewee: No problem, no problem

Interviewer: Thank you so much. Now, the first question goes like this. Can you tell us briefly the way you perceive your work? Are you happy with it? Is it really tiresome? Are there any challenges? Do you sometimes have to rash and work very quickly in order to clear the line? Feel free and tell us how it is.

Interviewee: I am happy with my job. It is more than ten years that I am doing it and if I was not happy with it I would have quitted. That is why I tell you that I am happy with it, no problem.

Interviewer: You don't meet any challenge? Don't you sometimes have to rash and work very quickly in order to clear the line?

Interviewee: Challenges are always there. You know that when you are based on a university hospital, you receive many cases. Most of the time we don't even go for break during lunch time because when you go, the patient is unhappy; they don't even realize that you have been working without rest, they only say that you abandon them and they can say that you offer a poor service. For patients, their only care is to be treated, they don't care about your health. That is why you keep working and go for break when you have cleared the line.

Interviewer: Were you expecting to receive such a great number of patients on a daily basis?

Interviewee: Yes, I expected it because day after day wherever I work, I have never seen a day with only few patients. It means that we have to work with courage since this is the job we applied for. I am happy with it but it is tiresome. However, it is rewarding because when you

treat a patient and see how he/she is happy and ask God to bless you (I even think that this is the reason why I am still doing it), this raise the morale.

Interviewer: This is really good. We also like this job. Now, tell us about giving oral health education to all patients who come to you. Tell us, is it really possible?

Interviewee: No, I don't want to lie to you. We don't have time for that. I know that this is important but we cannot get the time. Apart from that, we cannot address all the problems that the patient has on a single day. A patient may be complaining about three teeth but you treat only one. You only deal with the chief complaint and fix a rendezvous for other problems so that you might be able to serve all of them.

Interviewer: It means that the reasons why you don't do is …

Interviewee: (interjecting) The big number of patients and the time. You understand that if I receive fifteen patients for example and start to do a comprehensive care for each one of them, I can work until 10:00pm and not even finish. So, we work progressively because patients are many and clinicians are few.

Interviewer: When you manage to give oral health education to some patients like one or two, which are the main topics do you tell them about?

Interviewee: I insist especially on oral hygiene. Sometimes patients come to you and you realize that they have some responsibility on the problems they have. You blame them and tell them that they have delayed to come for treatment or that they don't brush their teeth correctly. When you check the oral hygiene you can realize that the patient never brushes the teeth. That is when you start educating them, asking how many times they brush their teeth per day, and what they use for brushing. We don't do it for all patients but sometimes you see that the patient really needs that.

Interviewer: Kindly feel free and tell us all you think.

Interviewee: Another thing, if a diabetic patient comes to you and you fail to tell them that they need a dental check up every six months, that they must brush their teeth correctly, what you did for them would be useless because they would be often coming back. When you give oral health education, it reduces the number of patients coming to you because they take preventive measures and don't develop the diseases.

Interviewer: When you give oral health education, do you have didactic materials or it is only in theory?

Interviewee: It is in theory. No didactic materials.

Interviewer: Now, tell us about scaling and polishing of teeth. Is it possible that you provide that treatment to every patient who need it?

Interviewee: No, we cannot do scaling and polishing for every patient who needs it. On my side, my plan is to do it for one patient per day. Currently we are in September but my appointments for scaling and polishing already reach October. However, the reason why I don't do more is because we have many patients and few scaling instruments. For patients with health insurances, who can afford the price or who need a quick service, I tell them that they can go to private clinics for dental cleaning instead of waiting the long list of appointments.

Interviewer: Are there many who need scaling and polishing?

Interviewee: Many, so many.

Interviewer: It means that the reason why you cannot do scaling and polishing for all of the patients as they come is lack of time and many patients. Is there any other reason?

Interviewee: Nothing more but even though I said that I give only one appointment per day, that concerns only people who need cleaning but who don't have periodontal diseases. When someone comes with a bad periodontal problem, I cannot fail to help him through scaling and polishing. When the chief complaint is related to dental cleaning, I do it without delay.

Interviewer: How many scaling can you perform per day, based on the number of instruments you have?

Interviewee: Like three patients but we can also sterilize when you see more people who need periodontal treatment. I can treat two patients then sterilize and treat more.

Interviewer: Tell us now about the sterilization of instruments. How is it?

Interviewee: No problem with the sterilization

Interviewer: Expand more on this

Interviewee: Sterilization? There is no problem about that because whenever I need sterile instruments I can get them. We have an autoclave in our service. There is no challenge about that. I can even sterilize myself but we also have a staff responsible for that. When she knows

that I want to do scaling for two patients, she collects used scaler tips from other wings, and then clean and sterilize them and when I need them she gives them to me. She is very young and active.

Interviewer: It means that you can never fail to treat a patient due to lack of a sterilized instrument?

Interviewee: Never (noise from the compressor)

Interviewer: Good. You told me that to give individual oral health education to patients is somehow impossible. What about discussing with the patient after treatment?

Interviewee: No, I don't. I only tell the patient that he/she needs an appointment. I also inform them in case they have other diseased teeth. In fact, you cannot miss to tell them about the next treatment, telling them that even if the carious teeth are not yet painful, they need to be filled, that they should come back for this treatment. I also tell them to brush their teeth correctly, reaching the back last teeth with the brush. I cannot pretend that I give a complete oral health education but we give some information.

Interviewer: That is also good, it is better than nothing. What about post-operative instructions? Are they given?

Interviewee: Yes, they are given. You cannot do an extraction and fail to give instructions; otherwise the patient would come back to you. If you fail to tell them not to spit and they go around spitting, they will come back to you with bleeding. We surely give post-operative instructions related to the treatment we did.

Interviewer: Now, in general, what about the quality of care that you provide? How is it? Are you satisfied with it or something should change?

Interviewee: We are professionals. I am happy with what I am providing, based on my scope of practice. I cannot speak for the others; I only speak for myself. The quality of care I provide is good. My conscious is telling me that I do a good work. That is demonstrated by the fact that I don't have patients coming back with treatment failure. That is why I say that at my level I provide good quality care. The biggest challenge we meet in public health facilities is linked to the tender process causing stock outs of consumables. You can see a cavity which you should fill with glass ionomer but since you don't have it, you put composite. You don't do it as well as you had to do it because you don't have the material which was required.

Interviewer: Good. Let us now talk about the available equipment. By equipment we mean like the dental chair, the sterilizer, the compressor and others. When one of them gets spoiled or is not functioning well, does the administration hurry up to repair it? Do they replace the spoiled piece without delay? How is it?

Interviewee: Yes, they care for the equipment. Our department has an appointed technician, a medical engineer charged of repairing dental equipment. He is based in the department of maintenance but when we call him, he immediately comes. He is skilled; not many things are difficult for him. Some of the items are still in the guaranty period but suppliers used to delay for coming to check the problem when we informed them. However, currently we partner with KIPHARMA. When something goes wrong on equipment provided by them which is still in the guaranty period, we write emails, copying all the levels including the head of maintenance, and they never exceed two to three hours without coming to check what happened. We have their emails; we write to the appointed personnel and give copies to the authorities. When he/she delays, they themselves ask why.

Interviewer: That is really good. Apart from the equipment, what about consumables like the polishing paste? What happens when they get finished?

Interviewee: Yes, they get finished because of the tender process as I told you before. At the beginning of the year, materials are there but currently we start to fall in stock outs. Toward the end of the year, many of the materials are finished. You can spend like three months without polishing. Currently we have the polishing paste, but I think that glass ionomer is not available. They tell you that the tender is undergoing but it can take even three months without receiving the needed material.

Interviewer: This is a challenge for sure. When you are doing treatments, do you feel secure especially about the risk of contracting an infectious disease? Do you trust your protection?

Interviewee: I don't understand well what you want to ask me

Interviewer: I am referring to the infection control

Interviewee: Infection control. I cannot say that we do it perfectly like a hundred per cent but personal protective equipment is there. One might fail to wear them but they are there.

Interviewer: Good. If they are available, it is ok. You have all it requires like the face masks, the gloves, the head caps, the eye protectors, everything?

Interviewee: All of them are available. Someone can forget to follow all the rules like washing hands before and after treatment, they can wash after treatment only but otherwise, everything is there.

Interviewer: Have you ever tried to request for those plastic covers for chair or light protection?

Interviewee: (hesitating). That question is beyond my ability; this is for the managers; maybe they did or they didn't but what I see is that we don't have them.

Interviewer: (smiling). That is ok. In research, participants have the right of not answering any given question as you have just done. Now, what could be done in order to ease your work in general?

Interviewee: If they would recruit more dental staff, this would make my job easier. I told you that I always work on pressure to clear the line. We need more staff, more chairs, the expansion of our clinic. We need to get to standards; if a dentist should treat five patients and for us we treat fifteen, you understand that we are still far from the standard ratios. I think WHO recommends a ratio of five patients by dentist, we need support. Another thing, if we think about the root of the problem, the reason why patients are many is that they are also many at district hospitals and the reason why they are many at the district hospital level is that they are many at health center level and the reason why they are many at health center level is that in the community they don't follow preventive measures. If people had knowledge on how to prevent dental diseases especially by brushing teeth and by going for check-up, we wouldn't have work to do. The root of the problem lies within the community; if I was a minister of health or had a post in the ministry of health, I would do a good job. I would start from the community and practice community dentistry. Otherwise, since they are so many at health center level, they also reach here in big numbers. That is the problem.

Interviewer: Is that the only reason or is there another reason why patients arrive here in great numbers?

Interviewee: Another reason is that there are some procedures that should be performed at district hospitals but which they don't do them due to lack of materials. However, we also know some of the dental practitioners of district hospitals who are lazy.

Interviewer: Really?

Interviewee: Yes.

Interviewer: What you said is very good and important about doing oral health education. That is related to what we were about to ask you next. As you said, community dentistry is really needed and it would have a positive impact by reducing the number of dental patients. If there was an application which would be installed in patients' smartphones in order to give oral health education in general, what impact that would have on your daily work?

Interviewee: That application would have a great impact on my daily work by reducing the patients number. If they get oral health knowledge through their smartphones, especially that what someone has in his/her smartphone is checked on very often, I would no longer receive many patients or patients whose cases have already been complicated. Most of the time we manage cases which are beyond the ability of other health facilities like cellulitis which were caused by extractions from the tradipractitioners. If people have that application, they would no longer go to those tradipractitioners. I don't know the content of that application but I think it would provide oral health education in general, guiding people when to go for dental check-up, why they should avoid going to tradipractitioners and other such things. I don't know how the owner of the application has designed it but I think that essentials topics would be there. It will help me by reducing the number of patients so that I might receive those I am able to manage without rushing.

Interviewer: Do you think that this application can reduce the time you used to spend with patients? What can you tell us about that?

Interviewee: Yes, it would reduce that time a lot. If I receive few patients, I would address all the problems they have and they would go home satisfied; but if they have a complaint on five teeth and I only treat one and send them home, it is a challenge. When the application will be there, patients will be informed and the number of patients will be reduced, so that I would have time for more patient's care.

Interviewer: Very good. Coming back to the dental materials, which advices can you give so that all the materials and equipment needed in teeth scaling and polishing are useful for you?

Interviewee: The advice I can give is that I should get quality materials and equipment I need, and on time. I don't know if I answer that question as I should.

Interviewer: It is ok, no problem. Now, which advices can you give in order to make your job easier in all domains?

Interviewee: So that I do my job comfortably?

Interviewer: Yes

Interviewee: First of all, I need to be able to work recommended hours by the government, instead of doing extra time; there are times we do extra working hours, when we have many patients. Secondly, I need motivation. I need salary increase because life is becoming very tough. When you have a salary which satisfies your needs, you are stable. We need salary and normal working hours.

Interviewer: What about the working environment? Are you satisfied with it? No challenge?

Interviewee: No problem, it is good.

Interviewer: It means that for you, you don't mind about working alone without an assistant?

Interviewee: That is what I was telling you that we need more staff but when they are not there you try to cope with the situation. You are your own assistant. Usually dentistry should be four-hands meaning that when you are working alone, you are not performing as you should.

Interviewer: Elsewhere currently they are now at six-hands dentistry

Interviewee: Six-hands! If they would ensure me four-hands dentistry, I would be satisfied.

Interviewer: Let them provide first four-hands dentistry and six-hands dentistry will come later.

Interviewee: Yes

Interviewer: Thank you so much, that is all we wanted to ask you. The information you gave us is very important and it will help us in this research we are doing. It will also be useful for the general dental health. Thank you.

Note taker: No more question, am satisfied and thank you for the time you gave us.

Interviewee: Thanks
