# Supplementary material for: Evaluation of oral health services and challenges faced by oral health practitioners working in Nyarugenge, Rwanda
Source: PLoS One. 2024 Aug 19;19(8):e0309127. doi: 10.1371/journal.pone.0309127 (PMC11332939; doi:10.1371/journal.pone.0309127)
Supplement: S1 Dataset — (ZIP) [file pone.0309127.s001.zip › dataset/Dataset qualitative interview transcript/PARTICIPANT (9).pdf]

INTERVIEW WITH PARTICIPANT 9

Interviewer: As we were telling you, we are conducting a PhD research about the challenges dental staff are meeting while treating Nyarugenge population but also the impact an application which would be put into the phone in educating patients about oral health would have on their work. In research there is no wrong answer, every answer is important. You also know that every information is kept with confidentiality, no one will know who said this or that. We would like that you answer freely and we are requesting your permission to record your answers so that we don't lose any information. Do you agree?

Interviewee: No problem

Interviewer: **Now for the first question, we would like that you tell us briefly how you feel about your work currently. If your job is tiresome, if you are pleased to do that job, if sometimes you have to rash and work very quickly in order to clear the line, if there are some challenges, feel free and tell us about how it is.**

Interviewee: Generally, I am happy with it but sometimes I am not happy due to the big number of patients so that you cannot satisfy all their needs. We can skip doing some treatments like scaling and dental fillings due to that number.

Interviewer: But otherwise you are happy with it?

Interviewee: Yes, I am happy with it, no problem.

Interviewer: Were you expecting to receive such a great number of patients on a daily basis?

Interviewee: No, I wasn't expecting that. I thought there would be no more than fifteen patients per day but currently I can receive thirty or even forty per day.

Interviewer: Sure? And how do you feel when you face such a situation? What effect does it have on you?

Interviewee: Since treating patients needs commitment, I accepted that situation.

Interviewer: Now, tell us about giving oral health education to all patients who come to you. Tell us, is it really possible?

Interviewee: It is not possible. I do it only for few among them. Some patients even make a request for education, while we had forgotten because of the big number of patients. You cannot do all the treatments and educate all the patients. Some patients ask questions on what to do in order to have healthy teeth.

Interviewer: Apart from the big number of patients, no other challenge for doing that?

Interviewee: Another thing is that in some of public institutions, they don't value scaling and fillings, they only value tooth extractions. Even availing materials for these treatments is not very important for the managers.

Interviewer: I was asking about giving oral health education to patients. The only reason why you don't do is the big number of patients or there are other challenges?

Interviewee: No other challenges. You only realize that if you start doing it, you would not have enough time for the treatments.

Interviewer: And when you manage to give oral health education to the patients who ask you questions, what are the main topics that you tell them about?

Interviewee: I focus on oral hygiene in general (some part is not audible)

Interviewer: When you are giving that oral health education, do you have didactic materials or you teach them only in theory?

Interviewee: Only in theory

Interviewer: Is it because the didactic materials are not there or it is because of time?

Interviewee: It is because didactic materials are not there

Interviewer: Now, tell us about scaling and polishing of teeth. Is it possible that you provide that treatment to every patient who needs it? Tell us about it.

Interviewee: No, it is not possible. We do scaling and root planning but polishing we don't do it. I have never done it here.

Interviewer: Is it because you don't have polishing materials?

Interviewee: Yes. You see, polishing procedure is not covered by community-based health insurance that is why polishing materials are not provided.

Interviewer: Are there many patients who need that treatment?

Interviewee: Yes, there are. I can say that one third of patients we receive need that.

Interviewer: They need dental scaling?

Interviewee: Yes

Interviewer: Tell us now about the sterilization of the scaling instruments. How is it at your health center?

Interviewee: Sterilization is really at a good level.

Interviewer: Explain us more so that we could understand what you mean by a good level (smiling)?

Interviewee: I am comparing with other areas, that is why I say that sterilization is at a very good level.

Interviewer: Yes, but we would like to know exactly how it is done? What kind of sterilizer do you have?

Interviewee: We use that sterilizer that doesn't use water, I don't remember the name

Interviewer: Dry heat sterilizer?

Interviewee: Yes, dry heat sterilizer

Interviewer: Is it located in your dental service so that whenever you need, you may sterilize?

Interviewee: Yes

Interviewer: It means that you can never fail to treat a patient due to lack of a sterilized instrument?

Interviewee: No, it never happened to me. It is possible that instruments finish but the one who helps you sterilizes in between and by the time instruments finish, others are ready.

Interviewer: How many patients for scaling can you treat per day, based on the number of instruments you have?

Interviewee: We can treat four patients.

Interviewer: Do you use ultrasonic scaler or manual scalers?

Interviewee: We use ultrasonic scaler.

Interviewer: How many scaler tips do you have?

Interviewee: We have four scaler tips

Interviewer: That is why you told me that you can do scaling for four patients?

Interviewee: Yes, but also because of patients who need other types of treatments and how strong we are.

Interviewer: You told me that it is challenging to give oral health education to patients. What about giving post-treatment instructions?

Inter*viewee: Yes, we do. Maybe not as comprehensively as it should be but we give them.*

Interviewer: When you think about the quality of care that you provide at your health center, how do you feel about it?

Interviewee: Usually the quality depends on the equipment and materials as well as on the practitioner. I can say that we try because our service is well equipped and patients are satisfied.

Interviewer: It means that you are able to deliver all the services, based on the package planned to be delivered at health center level?

Interviewee: Yes, we deliver them

Interviewer: When one of the equipment gets damaged like the dental chair, the sterilizer, and the compressor, those important machines like x-ray's; How does the administration react?

Interviewee: They understand and call for the technician.

Interviewer: Do they do it quickly, without delay?

Interviewee: Maybe not that same day, they can do it the following day but you can never say that they have neglected to do the repair.

Interviewer: **What about consumables like the polishing paste even though you told me that you never do polishing.**

Interviewee: As I told you at the beginning, the only consumables they care to avail are those linked to tooth extraction. But for the others, the administration is reluctant.

Interviewer: When you are doing treatments, do you feel secure especially about the risk of contracting an infectious disease?

Interviewee: Yes, because we have all the required equipment for self-protection.

Interviewer: Sure? Which ones do you have exactly?

Interviewee: We have medical coats, we have gloves, we have different dustbins and others (some items are not audible).

Interviewer: Do you have something to protect your eyes?

Interviewee: We have eye goggles

Interviewer: What about head caps?

Interviewee: No, we don't have

Interviewer: Yes. But in general you have enough personal protective equipment?

Interviewee: What is essential is there

Interviewer: Now, what could be done in order to ease your work in general?

Interviewee: If it was possible, recruiting another dental staff would be good even though it is challenging at the health center. Patients are many because they don't need reference processes, they come straight away. Another thing would be to avail needed consumables. In that case we would be really happy while offering services. In addition, the practitioner should have motivation.

Interviewer: Thank you so much. Now, if there was an application which would be installed in patients' telephones in order to give oral health education in general, what impact that would have on your daily work?

Interviewee: I don't know the way it would be installed and how it would work

Interviewer: It would be installed in smartphones like WhatsApp, you tube and others and it would provide oral health education through videos

Interviewee: The way it could help people like us who are in institutions is that since we have many patients, whenever a new education topic is out, we can forward it. Education materials in the application would be better prepared and they would provide more insight to the one watching them.

Interviewer: Do you think that this application can reduce the time you used to spend with patients?

Interviewee: On the number of patients we received?

Interviewer: No, on the time you spent teaching them.

Interviewee: Yes, the time would be reduced and also the stress of arriving and finding a lot of patients waiting for you. The impact would be great because you would no longer waste time.

Interviewer: Thank you so much. Now, which advices can you give so that all the materials and equipment needed in teeth scaling and polishing are useful for you?

Interviewee: I don't understand well the question.

Interviewer: Which advices can you give so that materials and equipment needed in dental cleaning in a dental setting are useful for you? So that you might be comfortable while using them?

Interviewee: First of all, these equipment and materials should be as valued as the ones for extractions. It requires that we keep negotiating with the administration.

Interviewer: Now, which advices can you give in order to make your job easier in general?

Interviewee: Even though the organigram plans only one dental therapist at the health center, it would be much better if another staff was added. Again, our authorities should allow us to take one day-off and rest.

Interviewer: Yes, rest is a necessity. Thank you so much, that is all we wanted to ask you.

Interviewee: Thank you too
