# Supplementary material for: Evaluation of oral health services and challenges faced by oral health practitioners working in Nyarugenge, Rwanda
Source: PLoS One. 2024 Aug 19;19(8):e0309127. doi: 10.1371/journal.pone.0309127 (PMC11332939; doi:10.1371/journal.pone.0309127)
Supplement: Attachment — Submitted filename: Response to reviewers.docx [file pone.0309127.s002.docx]

**RESPONSES TO THE EDITOR AND REVIEWERS PLOS ONE: ID: PONE-D-23-33199**

**EMMANUEL NZABONIMANA**

| Editor’s Comments | Responses | Section/page |
| --- | --- | --- |
| 1. Please ensure that your manuscript meets PLOS ONE's style requirements, including those for file naming. The PLOS ONE style templates can be found at  <https://journals.plos.org/plosone/s/file?id=wjVg/PLOSOne_formatting_sample_main_body.pdf>. and  <https://journals.plos.org/plosone/s/file?id=ba62/PLOSOne_formatting_sample_title_authors_affiliations.pdf> | RE: Entire manuscript verified and meets plosOne style requirement. |  |
| 2. Note from Emily Chenette, Editor in Chief of PLOS ONE, and Iain Hrynaszkiewicz, Director of Open Research Solutions at PLOS: Did you know that depositing data in a repository is associated with up to a 25% citation advantage (https://doi.org/10.1371/journal.pone.0230416)? If you’ve not already done so, consider depositing your raw data in a repository to ensure your work is read, appreciated and cited by the largest possible audience. You’ll also earn an Accessible Data icon on your published paper if you deposit your data in any participating repository (<https://plos.org/open-science/open-data/#accessible-data>). | All relevant data are within the manuscript and its supporting information files |  |
| 3. Thank you for stating the following financial disclosure:  "“EN was supported by the Consortium for Advanced Research Training in Africa (CARTA). CARTA is jointly led by the African Population and Health Research Center and the University of the Witwatersrand and funded by the Carnegie Corporation of New York (Grant No. G-19-57145), Sida (Grant No:54100113), Uppsala Monitoring Center, Norwegian Agency for Development Cooperation (Norad), and by the Wellcome Trust [reference no. 107768/Z/15/Z] and the UK Foreign, Commonwealth & Development Office, with support from the Developing Excellence in Leadership, Training and Science in Africa (DELTAS Africa) programme. The statements made and views expressed are solely the responsibility of the Fellow”"  Please state what role the funders took in the study.  If the funders had no role, please state: ""The funders had no role in study design, data collection and analysis, decision to publish, or preparation of the manuscript.""  If this statement is not correct you must amend it as needed.  Please include this amended Role of Funder statement in your cover letter; we will change the online submission form on your behalf. | RE: "The funders had no role in study design, data collection and analysis, decision to publish, or preparation of the manuscript." |  |
| 4. In the online submission form, you indicated that "Data are available upon reasonable request from the corresponding authors."   All PLOS journals now require all data underlying the findings described in their manuscript to be freely available to other researchers, either 1. In a public repository, 2. Within the manuscript itself, or 3. Uploaded as supplementary information. This policy applies to all data except where public deposition would breach compliance with the protocol approved by your research ethics board. If your data cannot be made publicly available for ethical or legal reasons (e.g., public availability would compromise patient privacy), please explain your reasons on resubmission and your exemption request will be escalated for approval. | RE: All relevant data are within the manuscript and its supporting information files. |  |
| 5. Check the number of questions you used in the interviews, you mentioned 13 but only 7 are described. | The number of questions have been checked and corrected. | Page 8-9 line 147-159 |
| 6. Mention the place of manufacturing of the sotfwares used in the study. | Manufacturing of the software used in the analysis had added (Stata manufactured by StataCorp LLC, USA and NVIVO manufactured by QSR International) | Page 9 line 171  Page 10, line 173 |
| 7. Use only one number after the point when you use percentages, otherwise do not use numbers after the point in percentages (there is not any precision here because of small frequencies). | One number after the point was used in all documents. |  |
| 8. Table 1, only should use frequencies, and probably summary a Total column with a frequency and percentage. | The frequency only was used in Table 1. | Page 12 line 215-216 |
| 9. The Tables are not very useful in this case, evaluate what tables really you need, you could summarize the information in a paragraph. | The Tables were deleted, and the information has been reported in a paragraph | Page 12 line 219-227  Page 13 line 231-236 |
| 10. Remove the box of the Discussion section | The box in the Discussion has been deleted. | Page 27, line 511-512 |
| Reviewer #1: |  |  |
| 11. This study is interesting and offer essential information. However, there are a few concerns:  Please check the grammar throughout the manuscript. In Title: (for example, it has to be '..challenges faced by..' instead of '..challenges facing..' | Comment well acknowledges.  The grammar throughout the manuscript has been checked and updated in the manuscript title updated to “Evaluation of oral health services and challenges faced by oral health practitioners working in Nyarugenge, Rwanda” | Page 1, Line 1 |
| 12. In introduction: '... This means public institutions are full of poor people...' Rephrase such sentences. | Sentence rephrased and updated in the revised manuscript  This suggests that public institutions are frequented by individuals who may be unable to afford costly dental treatments. | Page 4-line 56-58 |
| 13. Why is the theme and description table repeated again in DISCUSSION section? | comment acknowledged, and table deleted | Page 27, line 511-512 |
| 14. Is the quantitative data that is measured using an observation checklist all-inclusive? The authors have not made a note / mentioned about the other necessary equipment / consumables required (eg: Restorative materials, airotors, etc.) | The quantitative data measured using the checklist is not all-inclusive. This has been highlighted as the limitation of the study. | Page 32 line 618-621 |
